# Age-Driven Physiologically Based Pharmacokinetic Modeling of Empagliflozin: Toward Precision Dosing in Youth

**DOI:** 10.1021/acsptsci.5c00772

**Published:** 2026-05-29

**Authors:** Gabriela Pereira Milhm, Fernanda de Lima Moreira, Bárbara de Azevedo Abrahim-Vieira

**Affiliations:** † Laboratory of Molecular Modeling & QSAR (ModMolQSAR), Faculty of Pharmacy, Federal University of Rio de Janeiro (UFRJ), Av. Carlos Chagas Filho, 373, bloco L subsolo, Cidade Universitária, Rio de Janeiro, Brazil 21941-902; ‡ Laboratory of Pharmacometrics, Faculty of Pharmacy, Federal University of Rio de Janeiro (UFRJ), Rio de Janeiro, Brazil 21941-902

**Keywords:** empagliflozin, type 2 diabetes mellitus, T2DM, PBPK (physiologically based pharmacokinetic model), SGLT2 (sodium-glucose cotransporter 2), pediatric

## Abstract

Type 2 diabetes mellitus (T2DM) is a multifactorial disease that affects both adults and children. Treating T2DM in children poses a challenge because of the difficulties in conducting clinical trials in this special population. Empagliflozin (EMPA) is an antidiabetic drug belonging to the class of sodium-glucose cotransporter 2 (SGLT2) inhibitors and reversibly inhibits glucose reabsorption in the proximal tubules of the kidneys. This drug is a great candidate for treating T2DM in children due to its action on diabetes and other effects such as cardio- and nephroprotection. Despite its therapeutic advantages, the most appropriate dose for the treatment of T2DM in children is still unknown. In this context, new methodological approaches (NAMs) are necessary to overcome these obstacles. Physiologically based pharmacokinetic modeling (PBPK) emerges as a promising and regulatory-accepted approach that can be used to predict the most suitable dose for children. Therefore, the aim of this study was to construct the PBPK model for the oral administration of EMPA in children with healthy and obese weights. Following a literature search, pharmacokinetic and physicochemical parameters of the drug, along with physiological characteristics from selected studies for validation, were obtained for the model development. A PBPK model of EMPA was developed and verified in the adult population by comparing the simulated plasma exposure to the observed data. After validation for healthy adults, the model was scaled for children with healthy and obese weights according to the WHO metrics. The parameters that showed the most significant changes were the *C*_max_ and AUC values. In children with healthy weights, *C*_max_ was 1.93 and 1.39 times higher in the 10–12 year-old and 13–14 year-old groups, respectively, compared to adults receiving a 10 mg dose. Similarly, AUC increased by 1.81 and 1.48 times in the 10–12 year-old and 13–14 year-old groups, respectively, among healthy children. In obese children aged 10–12, *C*_max_ and AUC were altered, showing increases of 1.77 and 1.81 times, respectively. The developed and validated empagliflozin PBPK model in adults provided a robust basis for extending research to pediatric populations. Simulations indicated the need for dose adjustments in pediatric patients, particularly for healthy children aged 10–14 and obese children aged 10–12. These adjustments are essential to minimizing adverse effects, ensuring the safe and effective use of empagliflozin in these age groups.

## Graphical abstract

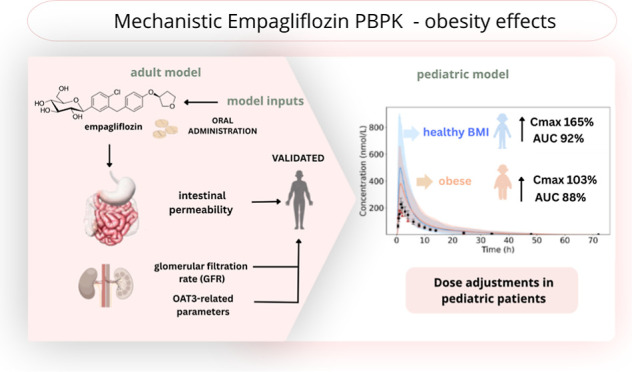

Type 2 Diabetes Mellitus (T2DM) is a chronic metabolic disorder characterized by hyperglycemia resulting from insulin resistance and/or insufficient insulin secretion by pancreatic β-cells.^1^ Insulin resistance is a core feature of both metabolic syndrome and T2DM,^2^ and its management is critical, as persistent hyperglycemia leads to micro- and macrovascular complications, including retinopathy, nephropathy, neuropathy, cardiovascular disease, and progressive β-cell destruction.^3^ Glycemic control remains the primary goal in the treatment of T2DM.^4,5^

T2DM is known as a disease with a worldwide prevalence and a tendency to increase each year. The 10th edition of the International Diabetes Federation atlas estimated that about 32 million individuals had T2DM in Latin America and 537 million worldwide in 2021.^6^ For the year 2045, estimates indicate that Latin America will have about 49 million people with T2D, while globally, this number will be 783 million.

In recent years, cases of T2DM in children and adolescents have increased considerably.^7–9^ It was estimated that in 2021 about 41,600 children and adolescents were diagnosed with T2DM worldwide.^9^ The risk factors for the development of this disease in young people are obesity, a sedentary lifestyle, and family history.^7,8,10,11^

In this age group, T2DM is accompanied by the presence of other comorbidities that characterize metabolic syndrome, and its prevalence is high in countries in South America, the Middle East, and the USA.^12,13^ Compared with adults, T2DM in children and adolescents appears more aggressive, with faster progression and higher long-term risk.^14,15^ Management is harder because ethical and safety constraints limit pediatric trials, resulting in fewer formally approved options.^16^

According to the American Diabetes Association and the European Association for the Study of Diabetes, therapeutic strategies aim to reduce blood glucose, body weight, and cardiovascular risk to prevent complications and slow disease progression.^17^ Regulatory alignment between major agencies (e.g., Food and Drug Administration (FDA)/European Medicines Agency (EMA)) centers on metformin and insulin as core therapies, with additional choices such as GLP-1 receptor agonists, semaglutide, liraglutide, dulaglutide, and exenatide ER approved for adolescents in some regions for pediatric T2DM treatment. However, most GLP-1 options are injectable, and metformin presents gastrointestinal side effects, which can affect adherence.^17,18^

SGLT2 inhibitors broaden the range of oral therapies for pediatric T2DM. In 2023, the FDA extended empagliflozin (EMPA) approval to children aged 10 years and older, adding a new option for those not adequately controlled with lifestyle and metformin. EMPA, an SGLT2 inhibitor first approved for adults in 2014, is available in 10 mg and 25 mg tablets; treatment typically starts at 10 mg and may be increased to 25 mg as needed.^19,20^ Mechanistically, SGLT2 inhibitors block sodium–glucose cotransport in the renal proximal tubules.

The kidneys reabsorb about 180 g of glucose daily, with SGLT2 responsible for roughly 90% of this flux.^21–23^ EMPA inhibition increases urinary glucose excretion by approximately 40–80 g/day, although partial reabsorption persists via compensatory SGLT1 upregulation.^22^ EMPA can be used as monotherapy when metformin is not tolerated or proves insufficient, a situation reported in a substantial proportion of patients, with estimates up to 50% in some sources.^17,24^ Its cardioprotective profile further supports its role in comprehensive diabetes care.^25^ Importantly, because SGLT2 inhibitors act through an insulin-independent mechanism, they can be combined with insulin sensitizers without overlapping pathways.

Given its efficacy, cardiometabolic benefits, and insulin-independent mechanism, empagliflozin (EMPA) is a strong candidate for pediatric type 2 diabetes mellitus (T2DM) therapy. However, the optimal pediatric dose remains unknown. Although clinical studies in adolescents and the recent FDA approval support its pediatric use,^26^ substantial variability in pharmacokinetic (PK) profiles has been observed across age groups, particularly between younger children and adolescents. These differences reflect developmental changes in physiology, metabolism, renal function, and body composition. Consequently, off-label use is often required but raises safety concerns, as pediatric PK parameters can differ markedly from those in adults, underscoring the urgent need for pediatric-specific drug development.^27–29^

In this context, physiologically based pharmacokinetic (PBPK) modeling has emerged as a key in silico strategy. By integrating drug-specific physicochemical properties with age-dependent physiological parameters, PBPK models simulate absorption, distribution, metabolism, and excretion (ADME) across virtual compartments representing organs and tissues.^30–32^ Compared with empirical or allometric approaches, PBPK provides superior mechanistic insight and more accurate predictions in younger populations, particularly for identifying sources of variability and extrapolating across subgroups.^33,34^ Its use has expanded globally, with regulatory agencies such as the U.S. FDA, the EMA, the Pharmaceuticals and Medical Devices Agency (PMDA), and the International Council for Harmonisation endorsing PBPK for pediatric extrapolation and risk assessment.^35–37^ The FDA’s pediatric extrapolation guidance (E11A), for example, explicitly recommends modeling and simulation to inform dose titration and trial design. This growing regulatory support reflects a paradigm shift toward model-informed decision-making, positioning PBPK as a powerful tool to optimize pediatric dosing, personalize therapy, streamline development, and reduce reliance on trials in vulnerable populations.

Therefore, the objective of this study is to develop and evaluate PBPK models of EMPA in adults and in pediatric populations with both healthy and obese body weights. Through these simulations, we aim to predict age-appropriate dosing, identify potential adjustments in therapeutic regimens, and provide evidence to support the rational, safe, and effective use of EMPA in children across different age groups.

## Methods

We developed a comprehensive whole-body PBPK model for EMPA using PK-Sim v.12, a software tool from the Open Systems Pharmacology Suite. The model construction incorporated the physicochemical and pharmacokinetic properties of EMPA, along with the physiological characteristics of healthy adults, as reported in selected literature sources. After inputting the parameters, simulations were performed and compared with clinical data from the selected studies as shown in Table S1 to validate the model. Once validated, the adult model was scaled to pediatric populations to enable the prediction and evaluation of appropriate EMPA dosing in children (Figure 1).

**Figure 1:**
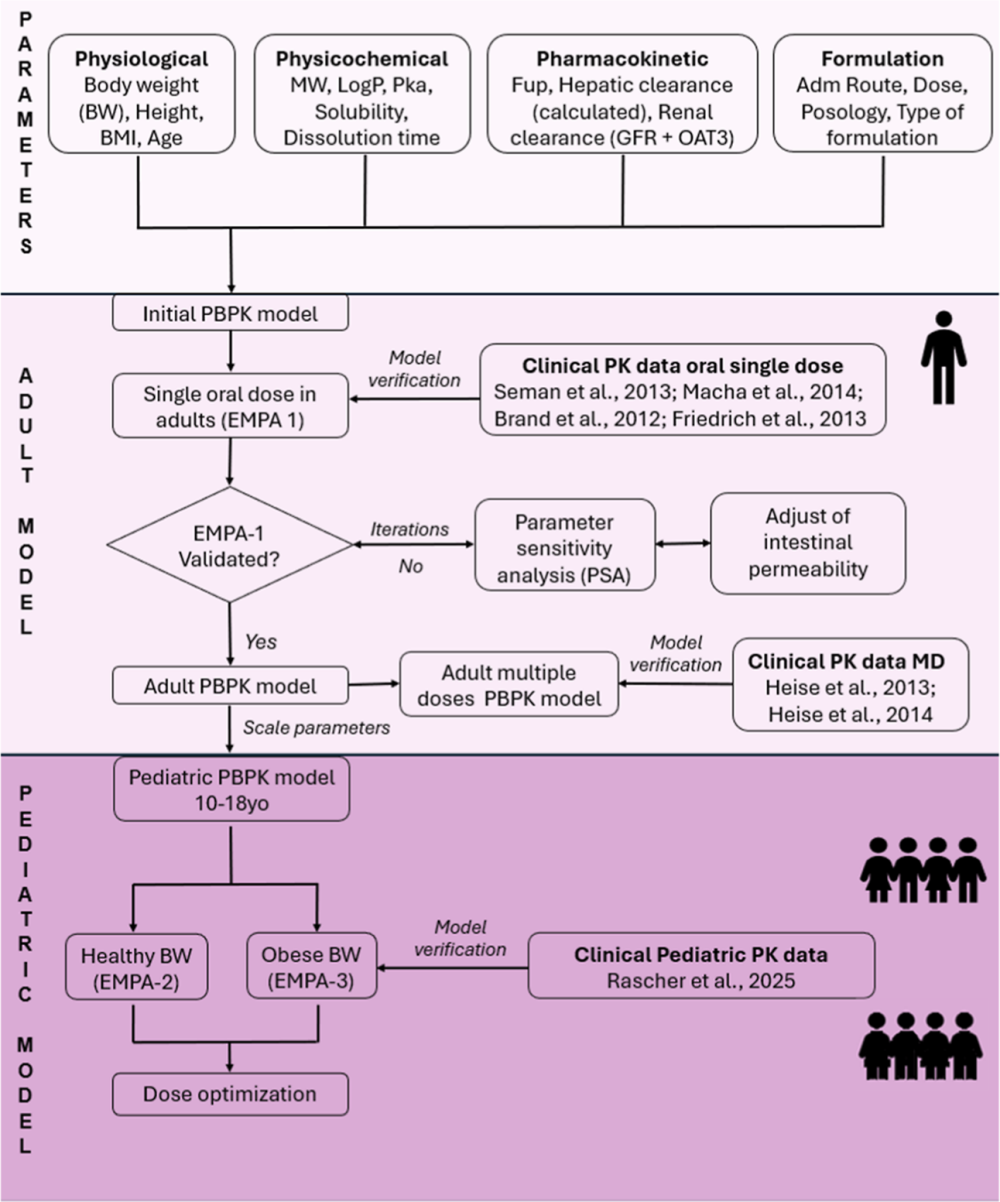
Workflow for PBPK model development and validation in adult and pediatric (10–18 years) populations.

### Software

PBPK modeling was performed using PK-Sim (version 12, Open Systems Pharmacology Suite, www.open-systems-pharmacology.org). PK-Sim provides a whole-body physiological framework, including organ volumes, blood flows, and age-dependent anatomical and physiological parameters, which were used to generate virtual populations and perform simulations. All simulations were conducted using the default human physiological database implemented in PK-Sim unless otherwise specified. Parameter identification, sensitivity analysis, and population simulations were performed using the built-in tools available in the PK-Sim environment.

### Development of the PBPK Model

The model was constructed using the middle-out approach, which combines elements from both top-down and bottom-up approaches.^38^ In this approach, available data from literature and clinical studies are utilized to estimate, during the model construction, other parameters that do not yet have defined values or when the value is uncertain.^39^

The development of the model was based on healthy adults with physiological parameters kept at default values provided by PK-Sim. During the construction and validation of the models, input data were used, which included physical-chemical and pharmacokinetic parameters of EMPA, formulation parameters, and anthropometric and physiological characteristics of the studied individual as described in Tables 1, S1, and S2.

**Table 1:** Physicochemical, Pharmacokinetic, and Formulation Input Data Used for Model Construction^a^

parameter	value	reference
Chemical formula	C23H27ClO7	PubChem
Molecular weight (g/mol)	450.91	PubChem
Log P (Log Units)	1.79	TGA, 2015 ^41^
p*K*_a_	p*K*_a_ (Strongest Acidic): 12.57	Drugbank
Dissolution time (50% dissolved)	5 min	Manoel et al., 2021 ^42^
Solubility (mg/mL)	0.40 at pH 6.8	FDA, 2014
Fraction unbound (%)	16.8	EMA, 2016 ^19^
Transcellular specific intestinal permeability (×10^–6^ cm/min)	2.41	Optimized
Administration route	Oral	FDA, 2014 ^20^
Partition coefficients model	Rodgers and Rowland	
Cellular permeabilities model	PK-Sim standard	
GFR fraction	1.0	FDA, 2014 ^20^
Total Hepatic Clearance	0.103 L/h/kg	
OAT3 expression	RT-PCR	Nishimura and Naito, 2005 ^43^
kcat OAT3 (min)	30	Ping; Wang; Gao, 2024 ^44^
OAT3 concentration (μM)	0.035	Wang et al., 2022 ^45^

a Legend: GFR, glomerular filtration rate; *k*_cat_, *V*_max_/transporter concentration; OAT3, Organic Anion Transporter 3; CLH, hepatic clearance; MW, molecular weight; p*K*_a_, acid dissociation constant; fup, fraction unbound in plasma. “Optimized” refers to parameters estimated during model calibration to best fit observed pharmacokinetic data.

As EMPA is primarily in a nonionized state at physiological pH, its absorption occurs through passive transcellular transport. This permeability is calculated based on physicochemical properties such as lipophilicity and effective molecular weight, using the standard permeability prediction model implemented in PK-Sim.^46–48^

The selection of the distribution model was guided by both the physicochemical properties of empagliflozin and an empirical performance evaluation. Considering its p*K*_a_ value of 12.57, empagliflozin exists predominantly in its nonionized form at physiological pH (∼7.4), with a calculated ionized fraction of approximately 6.8 × 10^–6^.^49^ This coexistence of states makes the Rodgers and Rowland model^46^ appropriate for predicting EMPA’s pharmacokinetic distribution. This is because the model is designed to account for both the ionized and nonionized forms of a compound, considering the electrostatic interactions that occur between the ionized drug components and anionic phospholipids at physiological pH.^40,85^

This theoretical rationale was confirmed through empirical validation during the parameter identification process, where several calculation methods for partition coefficients were compared. The Rodgers and Rowland method consistently provided the best fit to the observed data, yielding the lowest total error of 0.47. In contrast, other methods resulted in a poorer fit, such as PK-Sim Standard (Total Error = 1.16), Schmitt (Total Error = 1.11), Berezhkovskiy (Total Error = 0.60), and Poulin and Theil (Total Error = 0.53). A detailed summary of this comparative analysis is presented in Figure S1 in the Supporting Information that provides the agreement between the predictions generated by the Rodgers and Rowland model and the experimental data. Given its theoretical basis for handling EMPA’s ionization characteristics and its demonstrated empirical performance in our simulations, the Rodgers and Rowland model was selected for all subsequent analyses.

Since EMPA is a substrate of the OAT3 transporter, which mediates its active tubular secretion, OAT3-related parameters were incorporated into the model based on a previous PBPK study. Parameters related to tissue expression of the OAT3 transporter were derived from the PK-Sim database, which compiles literature-reported values. When multiple data sources were available, values based on quantitative RT-PCR measurements^43^ were prioritized, as they provide sensitive and gene-specific quantification of mRNA expression levels, enabling more consistent parametrization across tissues. Although the general renal clearance calculation retained glomerular filtration rate (GFR) fixed at 1, inclusion of OAT3 enabled a mechanistic representation of active tubular secretion. This was critical because glomerular filtration alone underestimated EMPA renal elimination; incorporating OAT3 improved the prediction of observed renal clearance and provided a more physiologically accurate description of the drug’s renal handling. By accounting for this transporter, the model captures both passive and active components of renal elimination, enhancing its predictive performance, particularly in scenarios such as renal impairment where active secretion may significantly contribute to overall clearance.^44,50^

Given the absence of clinical trials involving intravenous administration of EMPA, the PBPK model was developed and calibrated using high-quality oral administration data from adult populations. High-quality data were defined as clinical studies providing clearly reported dose information, plasma concentration–time profiles, subject characteristics, and sufficient sampling to adequately describe the pharmacokinetic profile, preferably obtained from peer-reviewed studies in healthy adults. The studies selected for model development and validation, as summarized in Table S1, included investigations in both healthy populations and patients with controlled type 2 diabetes mellitus patients. These comprised Phase I–II trials in healthy adults, evaluating oral doses ranging from 0.5 mg to 400 mg in single regimens.^51–53^ The data set was expanded with studies in controlled type 2 diabetes mellitus patients, including Macha et al. (2014) in renal/hepatic impairment subjects and two Heise et al. (2013) studies: 28 day multiple-dose (Heise et al. (2013)—MD 4W) and 8 day multiple rising dose (Heise et al. (2013)—MRD) protocols.^54–57^

### Simulation and Model Verification

After the insertion of physicochemical, pharmacokinetic, and physiological parameters, initial simulations were performed using the data sets from the studies employed for model development before obtaining the final model, which was achieved through iterative refinement and validation.

The training data set included plasma concentration–time profiles from healthy volunteers receiving single oral doses of 2.5, 10, and 50 mg (Seman et al., 2013) and a 50 mg dose in the renal impairment arm of Macha et al. (2014), selected for their comprehensive demographic and pharmacokinetic data.^53,57^ Plasma concentrations were estimated after completion of each simulation. The concentration–time profiles were analyzed during this internal validation, as well as the pharmacokinetic parameters *C*_max_, *T*_max_, and AUC. Sensitivity analysis and parameter identification tools were used to determine which input variables most strongly influenced the parameters under investigation and to optimize their values for alignment with clinical data.

After model optimization using the individual subject defined according to each clinical study, an additional simulation was performed by using a virtual population of 100 subjects based on the physiological characteristics reported for the study participants. This simulation was also validated against the data from corresponding publications. The virtual population, composed of 100 individuals, was generated with demographic and physiological characteristics modeled according to the information reported in each article selected for model development and validation. Simulations were then conducted using this virtual population, reflecting the physiological profiles described in the respective clinical trials, and the results were subsequently validated on the basis of the observed data extracted from the corresponding publications.

### Validation

Following development, the model’s predictive performance and external validation were evaluated against a diverse set of independent studies not used for training. This validation data set encompassed single- and multiple-dose conditions (ranging from 0.5 to 400 mg) from Brand et al. (2012), Friedrich et al. (2013), the hepatic impairment arm of Macha et al. (2014), additional doses from Seman et al. (2013), and multiple rising dose and four-week regimens from Heise et al. (2013).^51–56^ This approach supported a robust qualification of the model’s capacity to simulate systemic exposure across varying clinical contexts provided in Table S1.

Data points were digitized using WebPlotDigitizer v.4.6 (https://automeris.io/WebPlotDigitizer/) and incorporated into PK-Sim. Validation was performed through visual inspection and by calculating predicted/observed ratios for pharmacokinetic parameters, such as AUC, *C*_max_, and *T*_max_. For a model to be considered adequately validated, observed data should fall within the 90% confidence interval of the simulated population, and predicted/observed ratios should range between 0.5 and 2.^35,58,59^ This approach ensured that the model was appropriately developed, refined, and calibrated, providing reliable simulations aligned with the observed pharmacokinetic profiles across the clinical studies.^59^

To assess variability, the mean *C*_max_ and its reported standard deviation from the selected studies were used to calculate the relative error as a percentage of the mean, as shown in Formula [Disp-formula eq1]. This relative error was then applied to all points of the concentration–time curve, generating upper and lower bounds that reflect the expected variability across the profile. This approach ensures consistent representation of observed variability throughout the pharmacokinetic profile, providing a realistic depiction of experimental data and the uncertainty associated with model predictions.

1 $$Relative\;error=\frac{Standard\;deviation}{Mean\;C_{\max}}\times 100$$

### Pediatric Scaling PBPK Models (EMPA-2 and 3)

After validation of the model in healthy adults, it was scaled for pediatric populations by adjusting relevant physiological and pharmacokinetic parameters. Simulations included children and adolescents aged 10–18 years, considering both healthy-weight individuals (EMPA-2) and those with obesity (EMPA-3), to assess potential variations in pharmacokinetics.

Single-dose oral simulations of empagliflozin at 5 mg, 7.5 mg, and 10 mg were performed for individual virtual subjects with physiological characteristics assigned according to age, sex, weight, height, and body mass index (BMI). These data were collected using WHO reference values for healthy children,^60^ as shown in Table S3. Weight and BMI for the obese children were calculated to achieve a BMI z-score as close to +2.15 as possible, a value reported in the Treatment Options for Type 2 Diabetes in Adolescents and Youth—TODAY study.^14^ As shown in Figure S2, across both sexes, the simulated BMI values fall within the upper range of the reference distributions and are consistent with established definitions of pediatric obesity (BMI ≥ 95th percentile for age and sex).^61^ Differences between WHO^60^ and CDC^62^ growth curves reflect distinct reference populations and methodologies, with CDC charts based on historical data. Overall, these results support the physiological plausibility of the simulated obese pediatric populations.

The obese population was generated by using the standard population generator available in PK-Sim. Following the definition of these inputs, PK-Sim automatically recalculated physiological parameters, including organ volumes, tissue masses, and blood flows, based on its built-in scaling algorithms, which rely on established relationships between anthropometry and human physiology.^48^ In this framework, changes in the body size are propagated to system-specific parameters.

No manual modifications to tissue composition (e.g., fat mass fraction) were applied. This approach is consistent with PBPK applications in obese populations, where physiological variability is commonly implemented through anthropometric covariates rather than explicit redefinition of tissue composition.^63–66^

For population-based simulations, age groups were divided into three categories based on comparable physiological characteristics: 10–12, 13–14, and 15–18 years, as summarized in Table 2. These demographic and physiological data served as the basis for constructing the virtual pediatric population and performing population-based pharmacokinetic simulations. Population simulations included 100 virtual subjects per age group, with an equal proportion of males and females (50%), allowing evaluation of variability in pharmacokinetic parameters across age, sex, and body composition categories.

**Table 2:** Demographic Input Data of Grouped Children and Adolescents^a^

age (years)	weight (kg)	height (m)	BMI (kg/m^2^)
**normal weight**			
10–12	31.14–41.15	1.38–1.51	16.4–18.0
13–14	44.29–50.05	1.56–1.60	18.2–19.6
15–18	56.55–67.29	1.69–1.76	19.8–21.7
**obese weight**			
10–12	41.8–58.91	1.38–1.51	21.95–25.77
13–14	62.15–72.01	1.56–1.60	25.54–28.20
15–18	79.40–93.13	1.69–1.76	27.8–30.03

a Legend: BMI = body mass index.

Hepatic and renal clearance values from the adult model were applied in the pediatric simulations. This approach is physiologically plausible because empagliflozin elimination occurs predominantly via renal and hepatic pathways, and age-related changes in organ volumes, GFR, tissue composition, and blood flows were accounted for in scaling. Ontogeny of the OAT3 transporter was considered in the model, although it did not significantly influence simulations, as OAT3 activity and expression reach full maturation before adolescence.^67^ Other physiological parameters, including organ volumes, were consistent with literature values (Table S4).^68–74^

## Results and Discussion

Previous PBPK models of empagliflozin have primarily focused on adult populations, with limited evaluation of special populations in which physiological changes may significantly affect drug disposition.^44,75,76^ In the present study, the model developed and verified in adults was scaled to pediatric subjects aged 10–18 years, allowing the investigation of pharmacokinetic differences associated with growth. In addition, the present work evaluated the impact of increased body weight by simulating populations with different body compositions, including obese subjects. Obesity is known to modify physiological parameters such as tissue distribution, organ size, and blood flow, which may influence drug exposure.^77^ By incorporating these factors within a mechanistic PBPK framework, the current model enabled the assessment of empagliflozin pharmacokinetics across age groups and body compositions. This combined evaluation of developmental physiology and obesity-related changes extends previously published empagliflozin PBPK models and provides additional insight into potential differences in systemic exposure in adolescents.

### Development and Performance Evaluation of PBPK EMPA-1 Model: Single and Multiple Oral Doses of EMPA in Adults

Due to a lack of experimental and IV data on the enzymatic processes involved in EMPA metabolism, the model was simplified by applying total hepatic clearance, calculated from reported total and renal clearance values. EMPA elimination is primarily mediated via renal and hepatic pathways, with no other significant routes reported. To derive hepatic clearance in the absence of intravenous pharmacokinetic data, an indirect estimation approach was employed in which total systemic clearance was partitioned into renal and hepatic components. Specifically, hepatic clearance (CL_hepatic) was backcalculated using the relationship CL_total = CL_renal + CL_hepatic, based on observed plasma concentration–time profiles (Table S2). This type of clearance estimation, where systemic and organ clearances are inferred from oral PK and urinary data in the absence of IV data, has precedent in PBPK modeling and pharmacokinetic practice.^26,58^ These approaches provide a physiologically plausible representation of hepatic elimination in PBPK models built from oral clinical data sets.

For renal clearance, a combined approach was used, incorporating both a normalization factor for the GFR and data from the OAT3 transporter. In PK-Sim, GFR can be adjusted, where a value of 1 represents the absence of active reabsorption or secretion, corresponding to a filtration rate of 120 mL/min as defined by the software.^48^ Values greater than 1 indicate active secretion, whereas values below 1 indicate reabsorption. Since EMPA is a substrate of the OAT3 transporter, which mediates its active tubular secretion,^50^ OAT3-related parameters were incorporated into the model based on a previous PBPK study.^44^

Individual simulations were performed for each clinical study, reproducing the physiological characteristics of the enrolled subjects. However, the predicted pharmacokinetic parameters, *C*_max_ and AUC, were initially underestimated compared to observed data. To investigate these discrepancies, parameter sensitivity analysis was carried out. Intestinal permeability (P_eff) and lipophilicity (log P) emerged as the most influential parameters, governing both the rate and extent of absorption. Therefore, P_eff was adjusted, as in silico methods are known to underestimate the absorption of highly permeable drugs,^33^ which improved the agreement between simulated and observed data.

Using the parameter identification tool available in the software, an intestinal permeability value of 2.41 × 10^–6^ cm/min was estimated. After refinement, the simulated profiles closely matched the data sets used for both internal and external validation Figure S3, enabling the PBPK model to successfully reproduce the observed concentration–time profiles in healthy subjects.

One of the approaches used to evaluate model performance was the comparison between observed and predicted plasma concentrations as shown in Figure 2. On the basis of this analysis, more than 90% of the concentration data points across different studies fell within the 2-fold range, indicating overall good agreement between predictions and observations.

**Figure 2:**
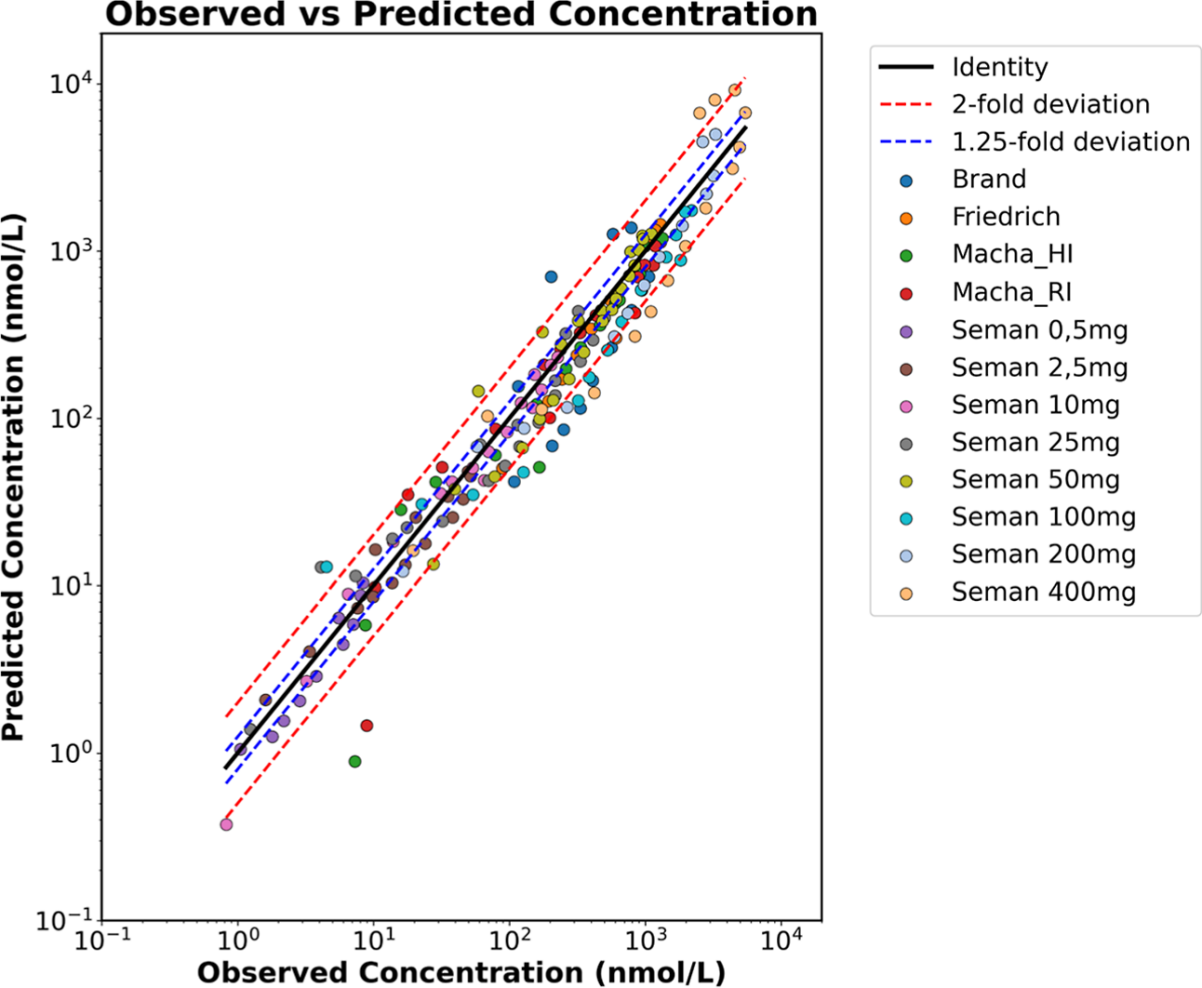
PBPK Model Performance of EMPA: Predicted vs Observed Plasma Concentrations. Observed versus PBPK-predicted plasma concentrations of EMPA across multiple studies and doses. The solid black line represents the line of identity (perfect prediction). Dashed red and blue lines indicate 2-fold and 1.25-fold deviation boundaries, respectively. Each data point corresponds to a mean concentration from a specific time point, study, and dose group.

Although a small proportion of points lie outside this interval, regulatory guidance does not define rigid acceptance criteria requiring all data to remain within a strict 2-fold range, and model evaluation is typically performed in a context-dependent manner.^37,58^ Importantly, none of the data points outside the 2-fold range correspond to clinically relevant doses of empagliflozin (10 and 25 mg), further supporting the adequacy of the model for its intended application.

It is also important to note that this analysis is based on concentration–time data across multiple sampling points, rather than solely on key PK parameters such as *C*_max_, AUC, and *T*_max_, which are more commonly used for assessing prediction accuracy. As a result, a greater degree of variability is expected in this type of point-by-point comparison.

Moreover, similar dispersion patterns, including data points outside the 2-fold range, have been reported in the PBPK literature, where models were still considered to have acceptable predictive performance.^78–81^ These deviations can be attributed to interindividual variability, experimental uncertainty, and limitations inherent to clinical data.

Population simulations were performed to account for interindividual pharmacokinetic variability. Figure 3 shows plasma concentration–time profiles from single-dose studies simulated using virtual cohorts of 100 individuals with physiological characteristics consistent with those reported in the original clinical trials. The predicted median curves (solid lines) were in close agreement with the observed data, while most measured values and their variability fell within the fifth–95th percentile prediction intervals (dashed lines), confirming the model’s ability to reproduce both central trends and variability across individuals.^32,33^

**Figure 3:**
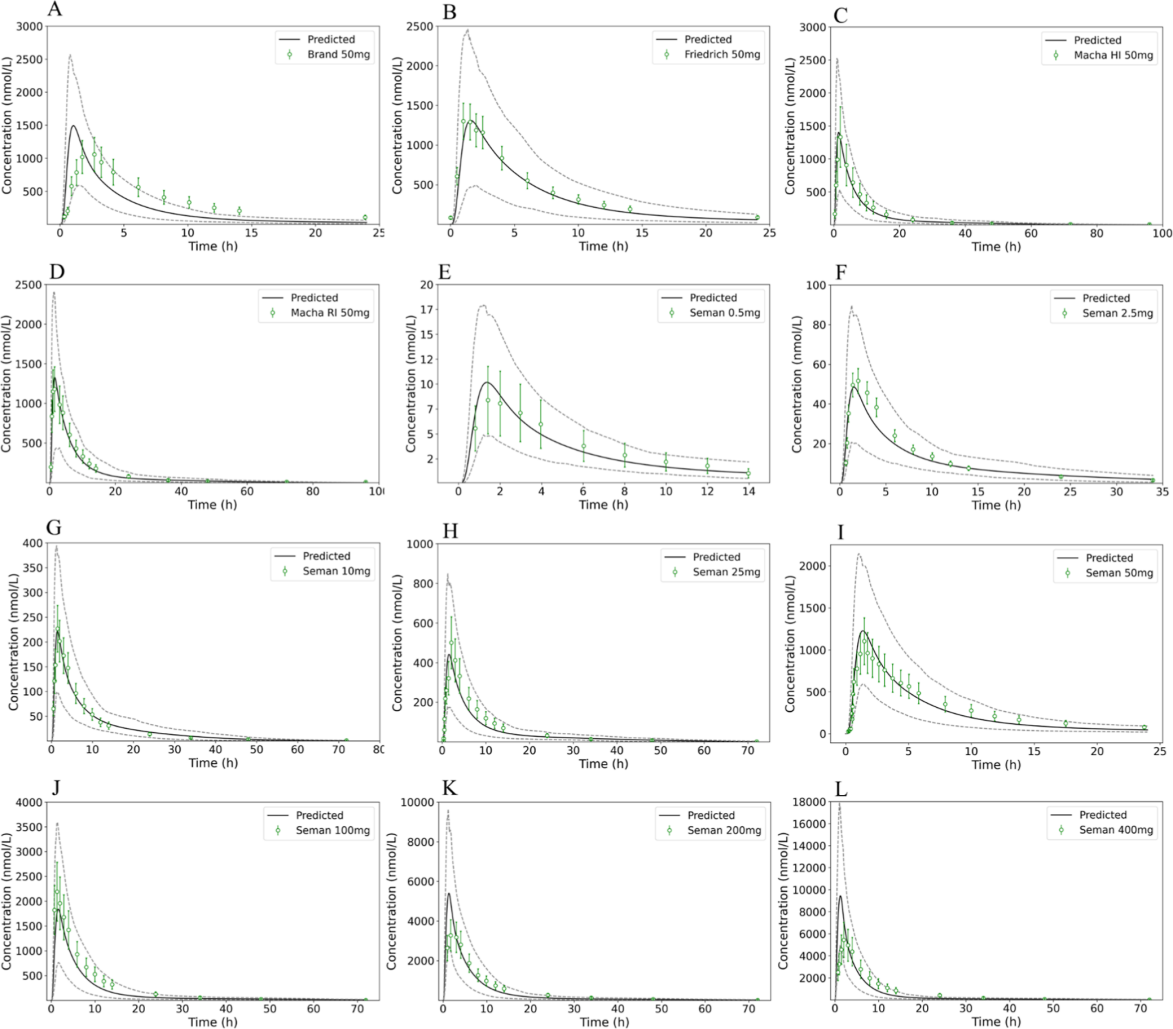
PBPK Model Performance for EMPA: Predicted vs Observed Concentration–Time Profiles across Studies and Doses. Green markers with error bars represent observed mean concentrations ± SD; solid black lines represent predicted medians, and dashed lines represent the fifth and 95th percentiles. Data from clinical studies are represented as (A) Brand et al., 50 mg; (B) Friedrich et al., 50 mg; (C) Macha et al. (RI), 50 mg; (D) Macha et al. (HI), 50 mg; (E) Seman et al., 0.5 mg; (F) Seman et al., 2.5 mg; (G) Seman et al., 10 mg; (H) Seman et al., 25 mg; (I) Seman et al., 50 mg; (J) Seman et al., 100 mg; (K) Seman et al., 200 mg; (L) Seman et al., 400 mg.

In virtual population simulations, predicted-to-observed ratios were close to unity for AUC (0.90), *C*_max_ (1.18), and *T*_max_ (1.05), with ranges of 0.70–1.05, 0.80–1.72, and 0.46–1.73, respectively (Table 3). Overall, these ratios, together with those obtained for multiple-dose scenarios, remained within the 0.5- to 2-fold range recommended in the literature and were generally close to unity, supporting the model’s predictive performance (Table S5). Simulated concentration–time profiles closely followed the observed data, capturing both central tendency and interindividual variability, as most experimental points fell within the prediction intervals (Figure 2).

**Table 3:** Comparison between the Pharmacokinetic Parameters Simulated in PK-Sim and Observed in Clinical Studies with a Single Dose^a^

article	dose (Route)	parameter	Obs	5%	95%	Pred	Pred/Obs
Brand et al., 2012	50 mg OD (healthy)	AUC_(0–*t*)_ (nmol·h/L)	8430 (20.9)	2662.97	9495.67	5917.38	0.70
		*C*_max_ (nmol/L)	1180 (23.8)	588.38	2605.04	1615.81	1.37
		*T*_max_ (h)	2.5 (1.0–4.0)	0.75	1.7	1.16	0.46
Friedrich et al., 2013	50 mg OD (healthy)	AUC_(0–*t*)_ (nmol·h/L)	9300 (12.5)	3492.18	14778.9	8215.18	0.88
		*C*_max_ (nmol/L)	1450 (17.1)	595.74	2547.11	1408.16	0.97
		*T*_max_ (h)	1.00 (1.00–3.00)	1.1	2.5	1.68	1.68
Macha et al., 2014—RI	50 mg OD (T2DM)	AUC_(0–inf)_ (nmol·h/L)	10,600 (16.4)	4327.5	16453.95	9642.59	0.91
		*C*_max_ (nmol/L)	1240 (23.5)	524.49	2579.87	1453.25	1.17
		*T*_max_ (h)	1.00 (1.00–3.00)	1	2.75	1.72	1.72
Macha et al., 2014—HI	50 mg OD (T2DM)	AUC_(0–inf)_ (nmol·h/L)	10,800 (22.6)	4149.36	16185.7	9856.08	0.91
		*C*_max_ (nmol/L)	1370 (33.9)	563.43	2574.41	1547.88	1.13
		*T*_max_ (h)	2.0 (1.0–4.0)	0.95	2.51	1.62	0.81
Seman et al., 2013	0.5 mg OD (healthy)	AUC_(0–inf)_ (nmol·h/L)	61.2 (17.2)	45.91	99.93	64.17	1.05
		*C*_max_ (nmol/L)	9.33 (3.73)	5.01	18.23	10.75	1.15
		*T*_max_ (h)	1.5 (1.0–3.0)	1	2	1.45	0.97
	2.5 mg OD (healthy)	AUC_(0–inf)_ (nmol·h/L)	396 (43.4)	259.39	626.7	391.62	0.99
		*C*_max_ (nmol/L)	53.2 (6.23)	21.16	92.37	51.32	0.96
		*T*_max_ (h)	1.8 (1.0–3.0)	1.1	2.25	1.64	0.91
	10 mg OD (healthy)	AUC_(0–inf)_ (nmol·h/L)	1730 (377)	1149.69	2865.22	1765.14	1.02
		*C*_max_ (nmol/L)	226 (46.0)	100.3	402.72	234.95	1.04
		*T*_max_ (h)	1.5 (1.0–2.0)	1.05	2.25	1.57	1.05
	25 mg OD (healthy)	AUC_(0–inf)_ (nmol·h/L)	3830 (825)	1628.43	5637.9	3155.18	0.82
		*C*_max_ (nmol/L)	505 (130)	183.25	877.39	468.92	0.93
		*T*_max_ (h)	2.1 (1.0–3.0)	1.15	2.25	1.67	0.80
	50 mg OD (healthy)	AUC_(0–inf)_ (nmol·h/L)	8580 (1680)	4120.17	12868.13	7676.28	0.89
		*C*_max_ (nmol/L)	1110 (274)	614.36	2168.74	1305.55	1.18
		*T*_max_ (h)	1.5 (0.8–3.0)	1	2	1.45	0.97
	100 mg OD (healthy)	AUC_(0–inf)_ (nmol·h/L)	16,500 (2390)	5223.72	23720.01	12017.36	0.73
		*C*_max_ (nmol/L)	2500 (666)	912.75	3977.59	1989.17	0.80
		*T*_max_ (h)	1.0 (0.8–3.0)	1.1	2.5	1.73	1.73
	200 mg OD (healthy)	AUC_(0–inf)_ (nmol·h/L)	31,200 (6260)	15175.4	51059.29	29214.56	0.94
		*C*_max_ (nmol/L)	3490 (816)	2913.67	10290.1	5913.21	1.69
		*T*_max_ (h)	1.8 (1.0–3.0)	0.95	2	1.43	0.79
	400 mg OD (healthy)	AUC_(0–inf)_ (nmol·h/L)	46,600 (10,200)	21712.72	72697.36	42078.16	0.90
		*C*_max_ (nmol/L)	6060 (1720)	5418.54	18931.28	10420.43	1.72
		*T*_max_ (h)	2.0 (0.8–4.0)	0.95	1.9	1.4	0.70

a OD: oral dose. Obs: observed; Pred: predicted.

This performance indicates that the model adequately represents variability across individuals and can anticipate pharmacokinetic behavior under real-world conditions, supporting its use for dose optimization and extrapolation to populations with different physiological characteristics including pediatric groups.

In multiple-dose scenarios (Figure S4), the simulated profiles showed good agreement with observed data, with prediction intervals encompassing the full range of interindividual variability. Predicted-to-observed ratios for AUC, *C*_max_, and *T*_max_ remained within the 0.5–2.0 acceptance range, supporting the robustness of the adult PBPK model.^59,82^ The model accurately reproduced the moderate accumulation behavior observed in both the 8 day and 4 week regimens, consistent with EMPA’s pharmacokinetic properties. Its relatively long terminal half-life (12–18 h) and moderate apparent volume of distribution (Vd ∼ 73–119 L) contribute to the observed accumulation until steady state is reached.^52,54,56,83^ The absence of excessive accumulation was consistent with the physicochemical properties of EMPA, and the model correctly predicted steady state within 72–96 h (4–5 half-lives), as expected for once-daily drugs.^51–54,56^ Simulated *C*_max_ concentrations aligned with observed variability, confirming the model’s reliability for predicting chronic exposure, particularly relevant for pediatric dose projections.

Other PBPK models of EMPA in adults have also been reported in the literature.^44,75,76^ A study conducted by Zhang and collaborators developed PBPK models for four SGLT2 inhibitors, including EMPA, to simulate and quantify drug distribution in target tissues and investigate inhibitory effects on SGLT1 and SGLT2 transporters.^75^ The models were built using GastroPlus software, and the EMPA model relied exclusively on a single clinical study by Sarashina et al., conducted in Japanese subjects across multiple dose levels.^84^ This limited data set may restrict the generalizability of the results to other populations. Compared to our model, some methodological differences are notable. While both approaches faced challenges in adequately representing drug absorption, our approach adjusted intestinal permeability, whereas the authors opted to optimize log *P*. In addition, like our model, they used estimated hepatic clearance values due to the absence of experimental data for EMPA metabolism.

Ping and colleagues developed a PBPK-UGE (Physiologically Based Pharmacokinetic–Urinary Glucose Excretion) model for EMPA to predict PK and UGE, as well as to investigate the compensatory role of SGLT1 in renal glucose reabsorption when SGLT2 is inhibited.^44^ Their model incorporated a Log *P* value similar to that used in our study, along with total hepatic clearance. A key feature of Ping et al.’s model was the inclusion of OAT3-mediated tubular secretion. The model included and optimized an OAT3 transport rate parameter (*k*_cat_), and sensitivity analysis confirmed its significant impact on UGE.

A more recent study by Guo et al. (2025) also developed a PBPK model for EMPA, constructing a physiologically based PK/PD model to predict EMPA behavior and optimize dosing in T2DM patients, including those at different stages of renal impairment.^76^ The model was implemented in PK-Sim and MoBi, with intestinal permeability optimized to improve absorption simulations, and hepatic clearance used to represent drug elimination. Some parameters differed from our model: Log *P* was set at 1.21 (lower than our value) and GFR fraction at 0.44, with no clarification provided for this GFR value.

Overall, the consistency observed across multiple studies, combined with the high proportion of data within the accepted range and the absence of any systematic trend toward over- or underprediction, supports the robustness and adequacy of the model for this work, as well as its reliability for future applications, such as scaling to pediatric populations or those with distinct physiological characteristics.^33,35,37,59^

### PBPK Models for Children and Adolescents: EMPA-2 and EMPA-3

Recently, EMPA was approved for pediatric use, based on the DINAMO clinical trial and the Office of Clinical Pharmacology (OCP) review, which represents a significant therapeutic advancement. However, a detailed analysis of these studies highlights a gap that can be addressed through physiologically based pharmacokinetic (PBPK) modeling, as developed in our work.^26,78^

After validation in healthy adults, the model was scaled to pediatric populations by adjusting the physiological and pharmacokinetic parameters. Simulations included children and adolescents aged 10 to 18 years, considering both healthy weight and obese individuals. Initial simulations were performed separately for each age, followed by grouping into three categories based on similar physiological characteristics to support the population-level analyses.

OAT3 ontogeny was considered in the model, although it did not significantly impact the simulations, as the transporter reaches full maturation in terms of activity and abundance before adolescence.^67^ Other parameters, including organ volumes and GFR, were also evaluated and showed values consistent with literature reports, confirming the physiological plausibility of the data obtained from the PK-Sim database (Table S4).^68–74^

Visual inspection of the simulated individual profiles indicated higher drug exposure in younger children, regardless of weight category, as shown in Figure 4. The highest exposure was observed in 10–13 year-old children, while adolescents closer to 18 years presented values more aligned with adult reference levels. Simulations for ages between 14 and 18 years were also consistent with observed pediatric data, supporting the external validation of the model, whereas younger ages showed greater deviation from these observations.

**Figure 4:**
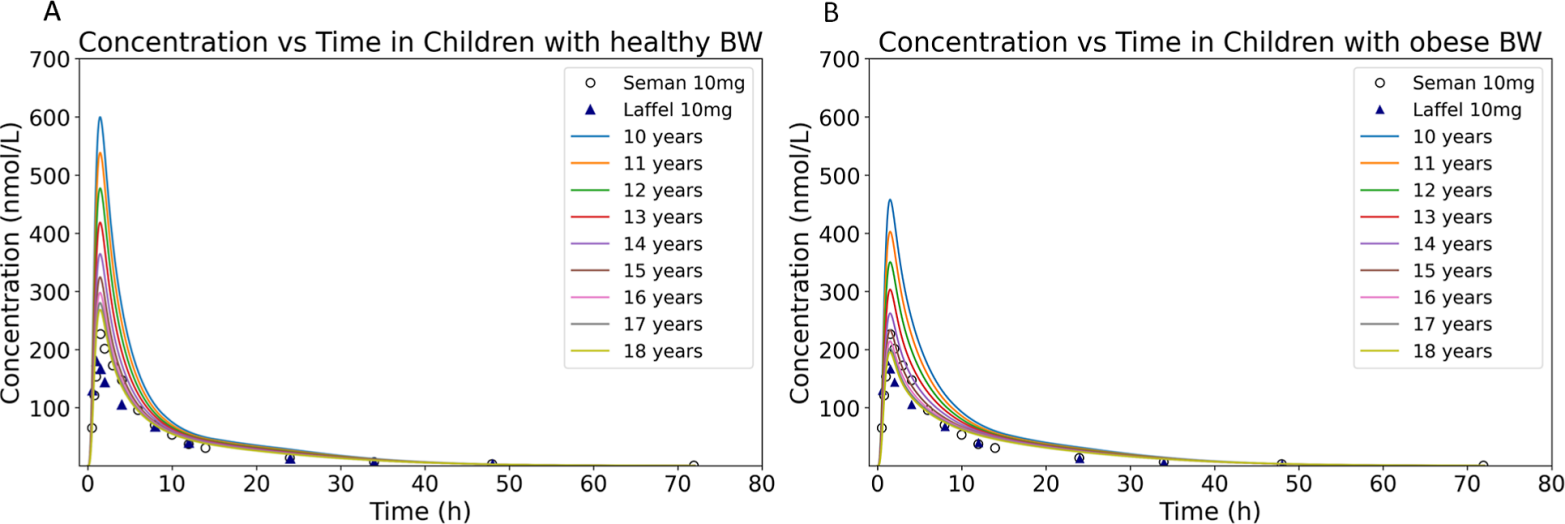
PBPK-simulated plasma concentration–time profiles of EMPA (10 mg) in children aged 10 to 18 years with healthy (A) and obese (B) body weight. Each line represents a specific age. Open circles indicate observed adult data from Seman et al. (2013) and pediatric data from Laffel et al., 2018 as blue triangles.

As shown in the individual simulations in Table S6, EMPA *C*_max_ values in children with healthy weight substantially exceeded the adult reference value (226 nmol/L), particularly between 10 and 13 years of age, with the highest exposure observed at 10 years, reaching up to 2.65 times the adult value. With increasing age, mean *C*_max_ values gradually decreased, approaching adult levels and reaching 1.19 times the reference at 18 years. Children with obesity consistently exhibited lower *C*_max_ values than their healthy-weight peers, suggesting that obesity may influence EMPA pharmacokinetics. The difference in *C*_max_ between weight groups decreased with age, from 141.6 nmol/L at 10 years to 73.0 nmol/L at 18 years, representing an average reduction of 105.25 nmol/L (∼27%) across the analyzed age range. From 16 years onward, *C*_max_ values in the obese group fell below the adult reference, decreasing further by 18 years.

To better reflect physiological similarities across developmental stages, children and adolescents were organized into three groups: Group I (10–12 years), Group II (13–14 years), and Group III (15–18 years), and simulations were performed using the virtual populations generated for each subgroup. The pharmacokinetic profiles for each group under healthy-weight and obese conditions are shown in Figure 5. In Group I, both healthy-weight and obese children showed higher exposure than adults, with predicted curves exceeding the upper bound of the confidence interval and remaining above observed adult and pediatric data. In contrast, for Groups II and III, simulated profiles were closer to observed values, with better overlap with both adult and pediatric data (mean age ∼14.5 years), supporting external validation of the model and indicating that exposure approaches adult levels with increasing age.

**Figure 5:**
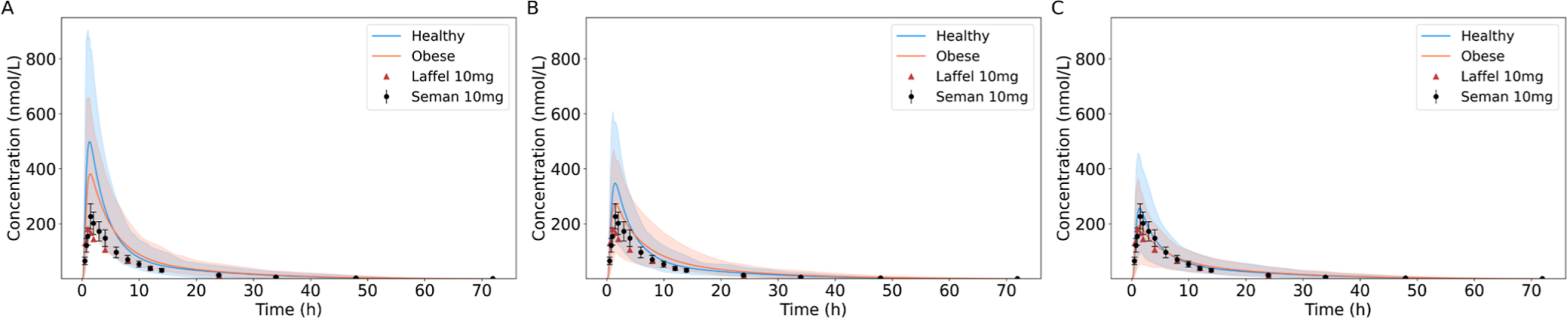
PBPK-predicted plasma concentration–time profiles of empagliflozin (EMPA) after a single 10 mg dose compared with observed clinical data. The panels show predictions for distinct pediatric age groups: (A) Group I (10–12 years), (B) Group II (13–14 years), and (C) Group III (15–18 years). Within each panel, solid blue and orange lines indicate the predicted median concentrations for healthy and obese subjects, respectively; the shaded areas represent the fifth and 95th percentiles of the prediction. Solid black symbols with error bars represent the observed mean concentrations ± SD from adult data (Seman et al., 2013) shown for reference and pediatric data (Laffel et al., 2018) as red triangles.

Table S7 summarizes the predicted pharmacokinetic parameters from pediatric population simulation, respectively. In healthy-weight children, mean AUC_inf_ was 1.80-, 1.40-, and 1.16-fold higher than in adults for Groups I–III, respectively, with slightly lower increases in obese children (1.73-, 1.38-, and 1.14-fold). Although exposure was higher across all pediatric groups, the magnitude of this difference decreased with age, particularly in individuals with obesity. *C*_max_ followed a similar age-dependent decline, with higher values in younger children and consistently lower levels in obese individuals. Compared to the adult reference, *C*_max_ increased 2.42-, 1.66-, and 1.21-fold in healthy-weight groups I–III, and 1.81-, 1.31-, and 0.93-fold in obese children, suggesting a more pronounced impact of body composition on peak concentrations than on overall exposure. *T*_max_ was not significantly affected by dose, age, or body composition. These findings indicate that body composition primarily influences *C*_max_, while age has a modest effect on AUC_inf_.

The DINAMO trial established EMPA efficacy but was conducted in a population with a mean age of approximately 14 years.^26,86,87^ The OCP review extrapolated these findings to the entire 10–17 year age range based on aggregated pharmacokinetic (PK) data indicating “comparable” exposure to adults. This approach raises important questions regarding dose optimization in a physiologically heterogeneous population. Differences in development between a 10 year-old and a 17 year-old, such as renal function, body mass, and metabolic maturation, can significantly influence pharmacokinetics. The study’s mean age may mask these variations, implying that a single-dose regimen (10 mg) may not be optimal for all individuals within the pediatric spectrum. Additionally, regulatory data show that subgroups such as lower-weight (<70 kg) and female patients exhibited higher concentrations than male or heavier patients.

Thus, while the DINAMO trial and OCP review establish the clinical efficacy of EMPA, our PBPK model provides a complementary methodology. It addresses the limitation of population heterogeneity in the study and offers a scientific basis for refining dosing strategies to ensure safer and more effective therapy for each patient within the pediatric age range.

On the basis of the finding that the standard 10 mg dose could lead to higher-than-reference exposure in certain pediatric profiles, the next step was to determine optimized dosing regimens. To this end, exploratory simulations were performed using alternative doses of 5 mg and 7.5 mg, in addition to the standard 10 mg, for each age subgroup and weight condition. Dose suitability was assessed through quantitative comparison of key pharmacokinetic parameters (AUC and *C*_max_) relative to adult reference exposure ranges, which serves as the established reference for drug safety and efficacy.^53,87^ Thus, for each pediatric subgroup, the dose that best aligned the systemic exposure with the adult target range was selected. This approach enables the proposal of dose optimization strategies aimed at achieving exposure profiles comparable to those observed in the adults.

In this study, adult exposure was used as a reference for dose evaluation, rather than implying a defined therapeutic exposure. This approach is supported by regulatory guidance and is commonly used in pediatric drug development when exposure–response relationships are not well established (FDA, 2020; EMA, 2018). Observed pediatric data, mainly from a population with a mean age of around 14 years, were used to evaluate model performance. Good agreement was seen for adolescents aged 13–14 years, while for other ages these data were used qualitatively to assess trends across the pediatric range. In contrast, dose evaluation was based on quantitative comparison with adult reference exposure (AUC and *C*_max_). These results support the use of exposure matching as a reasonable approach for dose evaluation. However, this relies on the assumption that disease progression and pharmacodynamic response are similar between adults and pediatric patients with type 2 diabetes. Although available evidence suggests general similarities, this remains a limitation, as exposure–response relationships were not evaluated in this study.

The pharmacokinetic data for EMPA presented in Figure 6 and Supporting Information Figures S5–S7, including AUC and *C*_max_ across different age groups (10–18 years), doses (5, 7.5, and 10 mg), and weight statuses (healthy-weight and obese), underscore the critical need for individualized dosing strategies. A general trend of decreasing EMPA exposure with increasing age was observed within the 10–18 year range. Additionally, for the same dose and age, obese individuals generally exhibited lower AUC and *C*_max_ values compared to their healthy-weight peers. These findings highlight the importance of adjusting EMPA dosing based on both age and weight status.

**Figure 6:**
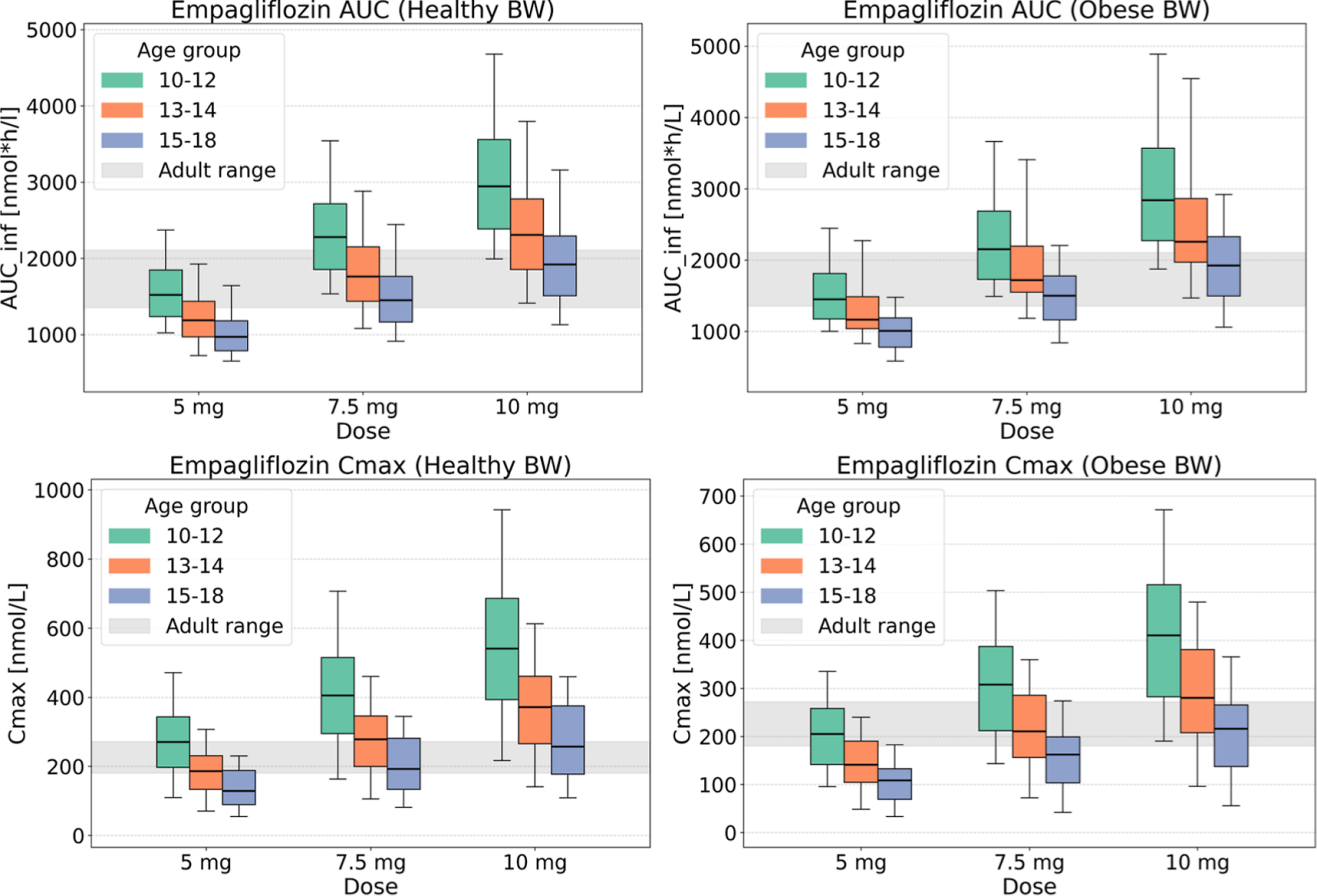
Box plots of PBPK-predicted empagliflozin AUC and *C*_max_ in pediatric populations (10–18 years), stratified by dose, age group, and body weight status. Boxes represent interquartile ranges with medians, whiskers indicate minimum and maximum values, and shaded regions represent observed adult reference ranges.

Based on this analysis, specific dose recommendations emerge for different age and weight subgroups. For younger children aged 10–12 years, a 5 mg dose appears more suitable for those of healthy weight, as it consistently yields exposure levels within or slightly above the adult reference range. However, for obese individuals within this same age, a 7.5 mg dose seems more suitable, aiming to counteract the diminished exposure often seen in this population and bring concentrations closer to the reference range. For adolescents aged 13–14 years, a 7.5 mg dose in healthy-weight individuals generally results in exposure within or near the adult reference range, whereas obese individuals in this age group may require a 10 mg dose to reach comparable exposure levels. Finally, in adolescents aged 15–18 years, the reduction in exposure with increasing age becomes more pronounced. For both healthy-weight and obese individuals in this group, a 10 mg dose appears necessary to achieve exposure within the adult reference range, as lower doses may result in values at or below this range.

Overall, these findings emphasize that age and obesity are important determinants of empagliflozin exposure in pediatric populations. Younger, healthy-weight children may experience higher-than-reference exposure, whereas older adolescents, particularly those with obesity, may require higher doses to achieve exposure comparable to that observed in adults. While these results provide mechanistic support for dose optimization, they should be interpreted in light of the absence of direct exposure–response data in this population.

## Conclusion

PBPK models of empagliflozin were developed for adults and extrapolated to children aged 10–18 years, enabling reliable dose predictions. A semimechanistic approach was used due to the complex metabolism and lack of kinetic data, providing a robust framework for simulations. Simulations highlighted the need for dose adjustments: younger healthy-weight children showed higher *C*_max_, while obese children had lower systemic exposure, emphasizing the impact of body composition over age alone. The model effectively captured age- and obesity-related pharmacokinetic differences, supporting individualized pediatric dosing for safe and effective EMPA therapy.

## Supplementary Material



## Data Availability

Data sharing is not applicable for this publication; no new clinical data were generated in this modeling study.

## Notes

Additional Supporting Information about materials, methods, and results, including demographic input data used for model development and validation; pharmacokinetic parameters from selected adult clinical studies; evaluation of tissue distribution models; optimization of the empagliflozin permeability parameter using observed plasma concentration–time data; and comparisons between simulated and observed pharmacokinetic parameters in adults and pediatric populations under single- and multiple-dose regimens; concentration–time profiles (linear and semilog), bar charts, and box plots of AUC and Cmax across ages, body weight conditions (healthy and obese), and doses; and simulated physiological parameters (e.g., organ volumes, hematocrit, body fat, and eGFR) for healthy-weight and obese virtual adult and pediatric populations (PDF)

## Abbreviations

AUC: Area Under the Plasma Concentration–Time Curve
BMI: Body Mass Index
BW: Body Weight
CL: Clearance
*C*_max_: Maximum Plasma, Concentration
EMA: European Medicines Agency
EMPA: Empagliflozin
FDA: Food and Drug Administration
GFR: Glomerular Filtration Rate
Log *P*: Logarithm of the Partition Coefficient
P_eff: Intestinal permeability
PBPK: Physiologically Based Pharmacokinetics
PK: Pharmacokinetics
p*K*_a_: Acid, Dissociation Constant
PSA: Parameter Sensitivity Analysis
RGR: renal glucose reabsorption
SGLT2: Sodium–Glucose Cotransporter 2
T2DM: Type 2 Diabetes Mellitus
*T*_max_: Time to Reach Cmax
UGE: Urinary Glucose Excretion
WHO: World Health Organization
