# Supplementary material for: Age-Driven Physiologically Based Pharmacokinetic Modeling of Empagliflozin: Toward Precision Dosing in Youth
Source: ACS Pharmacol Transl Sci. 2026 May 29;9(6):1412–25. doi: 10.1021/acsptsci.5c00772 (PMC13270446; doi:10.1021/acsptsci.5c00772)
Supplement: Supplementary file 1 [file pt5c00772_si_001.pdf]

Supporting Information

Age-Driven Physiologically-Based Pharmacokinetic Modeling of Empagliflozin: Toward Precision Dosing in Youth

Gabriela Pereira Milhm[1]; Fernanda de Lima Moreira[2]; Bárbara de Azevedo Abrahim-Vieira[1]*

[1] Laboratory of Molecular Modeling & QSAR (ModMolQSAR), Faculty of Pharmacy, Federal University of Rio de Janeiro (UFRJ), Av. Carlos Chagas Filho, 373, bloco L subsolo, Cidade Universitária, Rio de Janeiro, RJ, Brazil, 21941-902
[2] Laboratory of Pharmacometrics, Faculty of Pharmacy, Federal University of Rio de Janeiro (UFRJ), Rio de Janeiro, RJ, Brazil, 21941-902
Corresponding author email: *barbaraabrahim@farmacia.ufrj.br*

Table of Contents

Table S1 — Demographic Input Data Extracted from Articles for the Construction and Validation of the EMPA-1 Model S-3

Table S2 — Pharmacokinetic parameters from selected adult clinical studies. S-4

Figure S1 — Comparison of different tissue distribution models describing empagliflozin concentration–time profiles. The top panel shows linear scale and the middle panel semi-log scale. Observed data are shown as black circles, and lines represent model predictions (Berezhkovskiy, PK-Sim Standard, Poulin and Theil, Rodgers and Rowland, and Schmitt). S-5

Table S3 — Demographic Input Data of Children and Adolescents Calculated from WHO S-6

Figure S2 — Simulated BMI values for virtual pediatric subjects compared with BMI-for-age reference curves from WHO (2007) and CDC (2000) for (A) boys and (B) girls. The simulated population (Z ≈ 2.15) is shown alongside percentile (P95 and P97) and z-score (Z = 2) curves. S-7

Table S4 — Simulated mean values for liver, kidney, and heart volumes, hematocrit, body fat volume, and estimated glomerular filtration rate (eGFR) in healthy-weight and obese virtual adult and pediatric populations........ S-8

Figure S3 — Optimization of the Empagliflozin Permeability Parameter by Fitting Simulated Plasma Concentration-Time Profiles to Observed Adult Clinical Data S-9

Table S5 — Comparison between the pharmacokinetic parameters simulated in PK-Sim and observed in clinical studies with multiple doses........ S-9

Figure S4 — PBPK-predicted plasma concentration–time profiles of EMPA under multiple-dose regimens, compared with observed clinical data from Heise et al. (2013). Panels A–D show the multiple rising dose (MRD) study with 2.5 mg, 10 mg, 25 mg, and 100 mg once daily (OD) for 8 days. Panels E–G show the 4-week study in patients with type 2 diabetes mellitus (T2DM) receiving 10 mg, 25 mg, and 100 mg OD. Solid black lines indicate predicted median concentrations; dashed lines represent 5th and 95th percentiles. Green symbols with error bars represent observed mean concentrations ± SD. S-11

Table S6 — Comparison of pharmacokinetic parameters from individual and population simulations of children and adolescents (10–18 years) with observed adult data from the Seman et al., 2013 study and laffel study with a 10 mg dose. S-12

Table S7 — Comparison of pharmacokinetic parameters from population simulations of grouped children and adolescents (using PK-Sim) with observed data from single-dose clinical studies in adults (Seman et al., 2013)..S-13

Figure S5 — Bar charts of EMPA AUC and Cmax, comparing individuals with healthy body weight and obesity in the 10-to-18-year age range. The top row of charts (AUC in 10-18-year-olds, Cmax in 10-18-year-olds) shows the individual values for each year of age, from 10 to 18. The bottom row of charts (AUC by age groups, Cmax by age groups) clusters this data into three age ranges: 10–12 years, 13–14 years, and 15–18 years. Dashed horizontal lines indicate observed reference values in adults (FDA, 2014). S-14

Figure S6 — AUC and Cmax across different ages (10-18 years) and dosages (5 mg, 7.5 mg, 10 mg), stratified by healthy and obese weight. The upper panels show AUC, while the lower panels present Cmax. Horizontal dashed lines indicate reference values in adults. S-15

Figure S7 — Box plots of Empagliflozin AUC_inf and Cmax by pediatric age groups (10-12, 13-14, 15-18 years) and adults, stratified by health status (Healthy, Obese, Adult). (A) AUC; (B) Cmax. S-16

Table S1 — Demographic Input Data Extracted from Articles for the Construction and Validation of the EMPA-1 Model

Study	Drug administration	N (% female)	Ethnicity, n (%)	Age (SD)	Body Weight (SD)	Height (SD)	BMI (kg/m2)
Brand et al., 2012	50 mg for 5 days (healthy)	5 (0%)	European	26.4 (19-33)	78.6 (66-93)	1.84 (173-196)	x
Friedrich et al., 2013	50 mg for 5 days (healthy)	6 (83.3%)	White (100%)	26 (22.8-29.2)	x	x	23.73 (21.28-26.18)
Macha et al., 2013 - RI	50 mg (T2DM)	4 (66%)	White (100%)	25.8 (20.6-31)	81.,7	x	23.1 (20.4-25.8)
Macha et al., 2013 - HI	50 mg (T2DM)	6 (50%)	White (100%)	25 (20.3-29.7)	75	x	23.15 (20.82-25.48)
Seman et al., 2013	0.5 – 400mg for 3 days (healthy)	6 (0%)	European	36.5 (23-49)	79 (64-107)	180.0 (1.68-1.93)	24.8 (20-30.0)
Heise et al., 2013 - MD 4W	10mg for 28 days (T2DM)	6 (100%)	White (100%)	25.8 (17.9-33.7)	x	x	21.38 (19.67-23.09)
	25mg for 28 days (T2DM)	6 (66.7%)	White (66.7%)	22.8 (19.6-26)	x	x	23.60 (21.07-26.13)
	100mg for 28 days (T2DM)	8 (50%)	White (100%)	55 (45-65)	89.4 (77.8-101)	174.1 (168.2-180.0)	29.40 (26.20-32.60)
	100mg for 28 days (T2DM)	8 (50%)	White (100%)	55 (45-65)	89.4 (77.8-101)	174.1 (168.2-180.0)	29.40 (26.20-32.60)
Heise et al., 2013 - MRD	2.5mg for 8 days (T2DM)	8 (62.5%)	White (100%)	57.0 (37–67)	100.1 (84.2–112.9)	1.77	31.9 (28.8–34.3)
	10mg for 8 days (T2DM)	29 (58.6%)	White (89.7%)	57.0 (33–66)	101.7 (71.5–122.8)	1.83	30.3 (25.3–39.2)
	25mg for 8 days (T2DM)	15 (40%)	White (86.7%)	58.0 (40–68)	91.1 (67.2–121.4)	1.70	31.5 (26.3–36.3)
	100mg for 8 days (T2DM)	14 (78.6%)	White (92.9%)	61.0 (50–68)	85.4 (71.2–100.1)	1.76	27.6 (23.9–32.0)

Legend: MD 4W: Multiple doses for 4 weeks; MRD: Multiple rising doses; T2DM: Type 2 Diabetes Mellitus

Table S2 — Pharmacokinetic parameters from selected adult clinical studies.

Study	Dose and Duration	Hepatic Clearance (L/h)	Body Weight (kg)	CL hep/kg (L/h/kg)	Renal Clearance (L/h)	Total Clearance (L/h)
Brand et al., 2012	50 mg OD for 5 days (healthy)	Not reported	–	–	Not reported	9.86
Friedrich et al., 2013	50 mg OD for 5 days (healthy)	Not reported	–	–	Not reported	8.94
Macha et al., 2013 - RI	50 mg OD (T2DM)	6.135	81.7	0.075	1.71	7.85
Macha et al., 2013 - HI	50 mg OD (T2DM)	5.978	75	0.08	1.722	7.7
Seman et al., 2013	0.5 mg OD for 3 days (healthy)	10.512	79	0.133	3.078	13.59
	2.5 mg OD for 3 days (healthy)	8.052	79	0.102	2.478	10.53
	10 mg OD for 3 days (healthy)	7.14	79	0.09	2.466	9.61
	25 mg OD for 3 days (healthy)	8.6	79	0.109	2.262	10.86
	50 mg OD for 3 days (healthy)	7.76	79	0.098	1.926	9.69
	100 mg OD for 3 days (healthy)	7.638	79	0.097	2.442	10.08
	200 mg OD for 3 days (healthy)	7.924	79	0.1	2.736	10.66
	400 mg OD for 3 days (healthy)	11.31	79	0.143	2.97	14.28
FDA Report	–	Not reported	–	–	Not reported	10.6
EMA Assessment	–	Not reported	–	–	Not reported	10.6
**Total Mean**	–	**8.105**	–	**0.103**	**2.379**	**9.91**

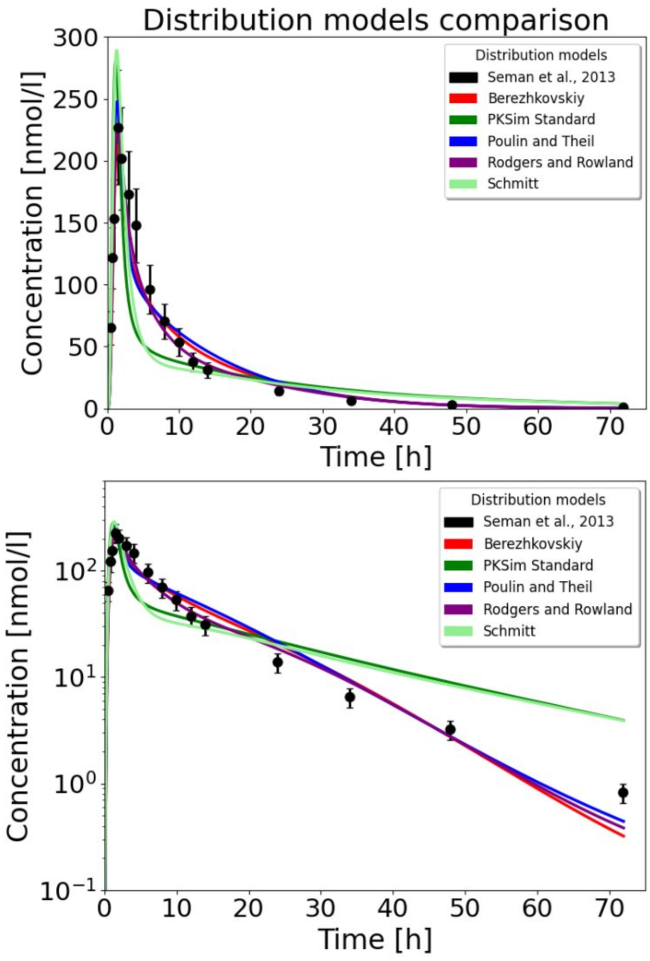

Run #	Description	Total Error
4	Rodgers and Rowland	0.47
2	PK-Sim Standard	1.16
1	Berezhkovskiy	0.60
5	Schmitt	1.11
3	Poulin and Theil	0.53

Figure S1 — Comparison of different tissue distribution models describing empagliflozin concentration–time profiles. The top panel shows linear scale and the middle panel semi-log scale. Observed data are shown as black circles, and lines represent model predictions (Berezhkovskiy, PK-Sim Standard, Poulin and Theil, Rodgers and Rowland, and Schmitt).

Table S3 — Demographic Input Data of Children and Adolescents Calculated from WHO

Normal weight				Obese weight (Z-score BMI = +2.15)			
Age (years)	Weight (Kg)	Height (m)	BMI (kg/m^2)	Age (years)	Weight (Kg)	Height (m)	BMI (kg/m^2)
10	31.14	1.38	16.4	10	41.80	1.378	21.95
11	34.61	1.43	16.9	11	47.28	1.431	23.09
12	38.90	1.49	17.5	12	54.02	1.491	24.3
13	44.29	1.56	18.2	13	62.15	1.560	25.54
14	50.61	1.63	19	14	71.19	1.632	26.73
15	56.55	1.69	19.8	15	79.40	1.690	27.8
16	61.28	1.73	20.5	16	85.80	1.729	28.7
17	64.77	1.75	21.1	17	90.37	1.752	29.44
18	67.29	1.76	21.7	18	93.13	1.761	30.03

Legend: BMI: Body Mass Index

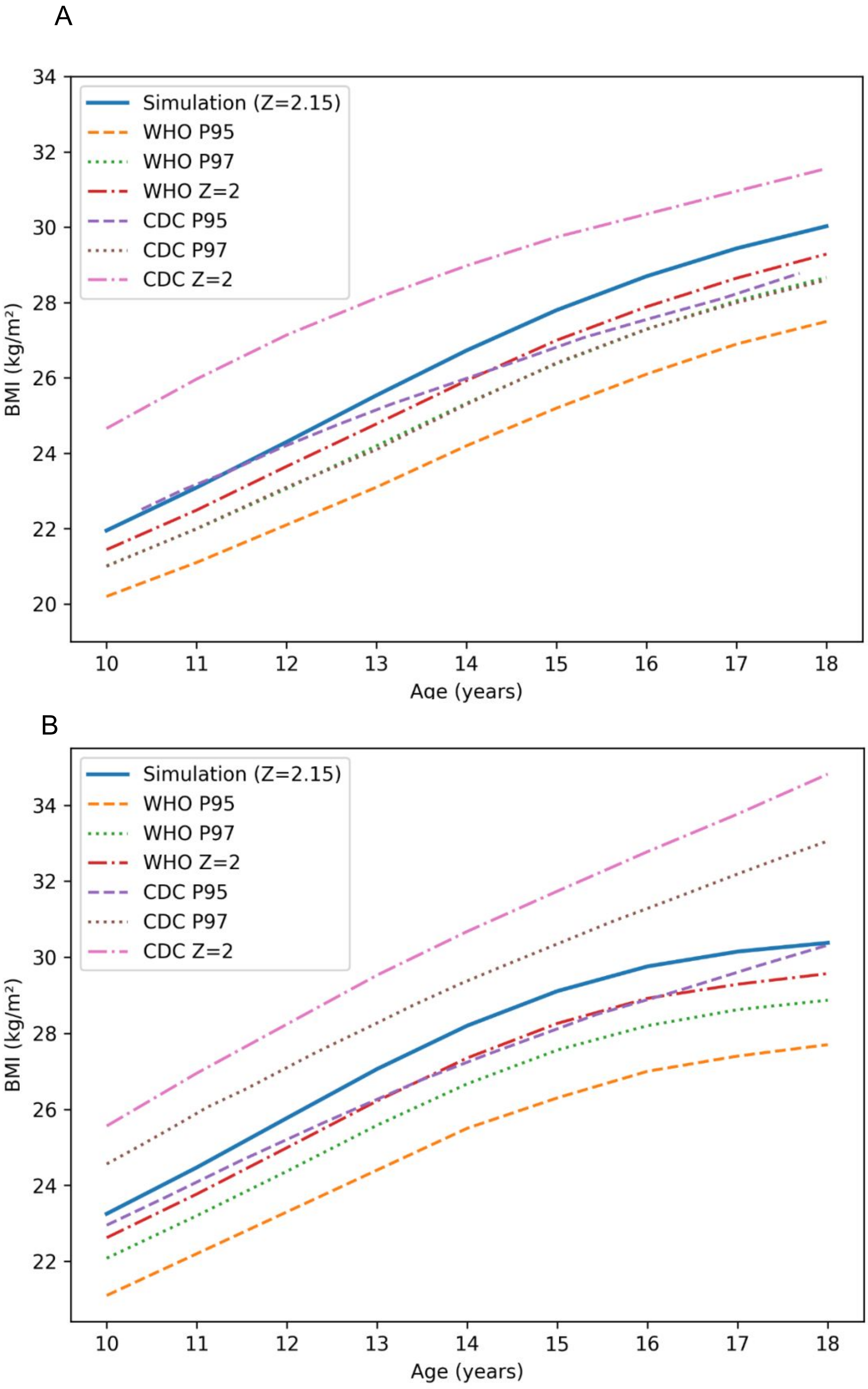

Figure S2 — Simulated BMI values for virtual pediatric subjects compared with BMI-for-age reference curves from WHO (2007) and CDC (2000) for (A) boys and (B) girls. The simulated population (Z ≈ 2.15) is shown alongside percentile (P95 and P97) and z-score (Z = 2) curves.

Table S4 — Simulated mean values for liver, kidney, and heart volumes, hematocrit, body fat volume, and estimated glomerular filtration rate (eGFR) in healthy-weight and obese virtual adult and pediatric populations.

Population	Age (years old)	Condition	Hematocrit	Liver volume (L)	Fat volume (L)	Heart volume (L)	Kidney volume (L)	eGFR (ml/min/1.73m²)	Reference
Children and Adolescent	10	Health	0.4	1.12	6.92	0.18	0.26	109.64	PK-Sim database
		Obese	0.4	1.14	16.92	0.18	0.26	95.09	
	11	Health	0.4	1.22	6.43	0.2	0.28	109.03	
		Obese	0.4	1.25	18.01	0.2	0.28	93.83	
	12	Health	0.4	1.34	6.49	0.22	0.3	107.53	
		Obese	0.4	1.38	20.03	0.23	0.3	91.92	
	13	Health	0.43	1.47	7.07	0.25	0.32	105.39	
		Obese	0.43	1.51	23	0.25	0.32	89.55	
	14	Health	0.43	1.61	8.1	0.27	0.34	102.89	
		Obese	0.43	1.64	26.63	0.27	0.34	87.22	
	15	Health	0.43	1.73	9.19	0.29	0.36	101.11	
		Obese	0.43	1.77	30.03	0.3	0.36	85.71	
	16	Health	0.43	1.81	11.01	0.31	0.37	99.04	
		Obese	0.43	1.84	33.92	0.31	0.37	84	
	17	Health	0.43	1.88	12.52	0.32	0.38	97.99	
		Obese	0.43	1.89	36.75	0.32	0.38	83.13	
	18	Health	0.43	1.93	13.85	0.33	0.38	97.45	
		Obese	0.43	1.94	39.52	0.33	0.39	83.01	
Adult	35	Health	0.47	2.39	19.66	0.43	0.46	104.88	Seman et al., 2013
	30	Obese	0.47	2.41	49.65	0.42	0.44	87.24	Virtual individual

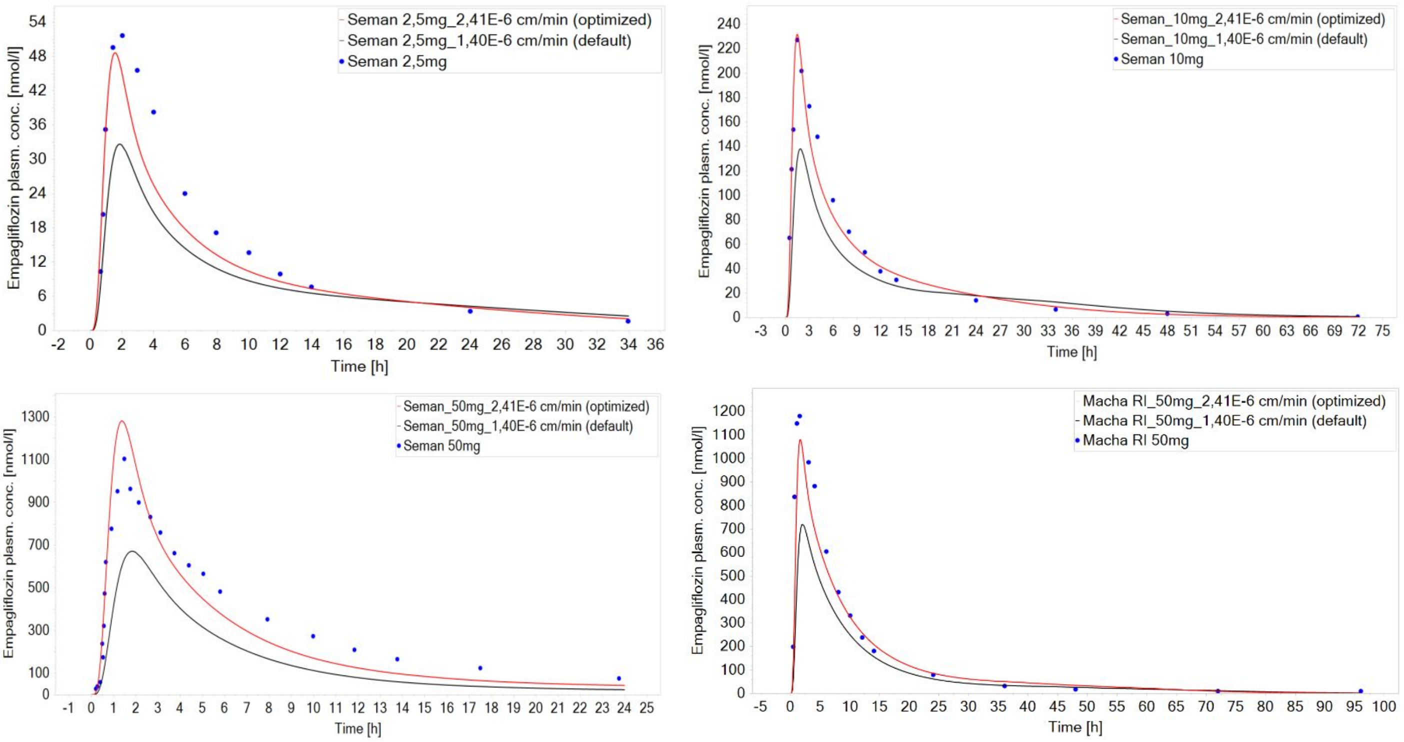

Figure S3 — Optimization of the Empagliflozin Permeability Parameter by Fitting Simulated Plasma Concentration-Time Profiles to Observed Adult Clinical Data

Table S5 — Comparison between the pharmacokinetic parameters simulated in PK-Sim and observed in clinical studies with multiple doses

Article	Dose (Route)	Parameter	Obs	Pred	Pred/Obs
Heise et al., 2013 - MD 4W	10 mg for 28 days (T2DM) – First dose	AUC(0-t) (nmol·h/L)	1550 (16.2)	1643.29	1.06
		Cmax (nmol/L)	309 (45.2)	202.96	0.66
		Tmax (h)	1.5 (1.0–2.5)	1.55	1.03
	25 mg for 28 days (T2DM) - First dose	AUC(0-t) (nmol·h/L)	3930 (22.9)	4039.79	1.03
		Cmax (nmol/L)	722 (20.0)	570.18	0.79
		Tmax (h)	1.5 (0.8–2.0)	1.45	0.97
	100 mg for 28 days (T2DM) - First dose	AUC(0-t) (nmol·h/L)	15900 (21.2)	11555.7	0.73
		Cmax (nmol/L)	2630 (25.8)	1751.53	0.67
		Tmax (h)	1.5 (0.8–3.0)	1.65	1.10
	10 mg for 28 days (T2DM) – Last dose	AUC(0-t) (nmol·h/L)	1870 (15.9)	1967.84	1.05
		Cmax (nmol/L)	259 (24.8)	229.12	0.88
		Tmax (h)	1.5 (1.0–4.0)	1.5	1.00
	25 mg for 28 days (T2DM) - Last dose	AUC(0-t) (nmol·h/L)	4740 (21.2)	4584.47	0.97
		Cmax (nmol/L)	687 (18.4)	617.75	0.90
		Tmax (h)	1.5 (0.8–3.0)	1.51	1.01
	100 mg for 28 days (T2DM) - Last dose	AUC(0-t) (nmol·h/L)	18 700 (25.2)	17641.64	0.94
		Cmax (nmol/L)	2390 (28.1)	1823.39	0.76
		Tmax (h)	1.5 (0.8–6.0)	1.75	1.17
Heise et al., 2013 - MRD	2.5mg for 8 days (T2DM) - First dose	AUC(0-t) (nmol·h/L)	402 (68)	473.39	1.18
		Cmax (nmol/L)	62.4 (12.3)	45.2	0.72
		Tmax (h)	1.5 (0.7–1.5)	1.45	0.97
	10 mg for 8 days (T2DM) - First dose	AUC(0-t) (nmol·h/L)	1,630 (231)	2129.34	1.31
		Cmax (nmol/L)	245 (51.5)	214.48	0.88
		Tmax (h)	1.5 (1.0–2.0)	1.45	0.97
	25 mg for 8 days (T2DM) - First dose	AUC(0-t) (nmol·h/L)	4,310 (1,040)	4039.79	0.94
		Cmax (nmol/L)	606 (147)	537.13	0.89
		Tmax (h)	1.5 (1.0–4.0)	1.55	1.03
	100 mg for 8 days (T2DM) - First dose	AUC(0-t) (nmol·h/L)	20,000 (3,640	19823.28	0.99
		Cmax (nmol/L)	2,750 (701)	2216.06	0.81
		Tmax (h)	3.0 (1.0–4.0)	1.68	0.56
	2.5 mg for 8 days (T2DM) - Last dose	AUC(0-t) (nmol·h/L)	471 (108)	474.13	1.01
		Cmax (nmol/L)	68.5 (16.8)	51.52	0.75
		Tmax (h)	1.5 (1.0–2.0)	1.5	1.00
	10 mg for 8 days (T2DM) - Last dose	AUC(0-t) (nmol·h/L)	2,030 (362)	2570.71	1.27
		Cmax (nmol/L)	283 (90.1)	241.66	0.85
		Tmax (h)	1.5 (1.0–2.0)	1.5	1.00
	25 mg for 8 days (T2DM) - Last dose	AUC(0-t) (nmol·h/L)	4,990 (1,080)	4584.47	0.92
		Cmax (nmol/L)	630 (106)	577.73	0.92
		Tmax (h)	2.0 (0.7–4.2	1.5	0.75
	100 mg for 8 days (T2DM) - Last dose	AUC(0-t) (nmol·h/L)	22,800 (5,700)	22386.25	0.98
		Cmax (nmol/L)	2,750 (605)	2358.46	0.86
		Tmax (h)	1.75 (1.0–4.0)	1.75	1.00

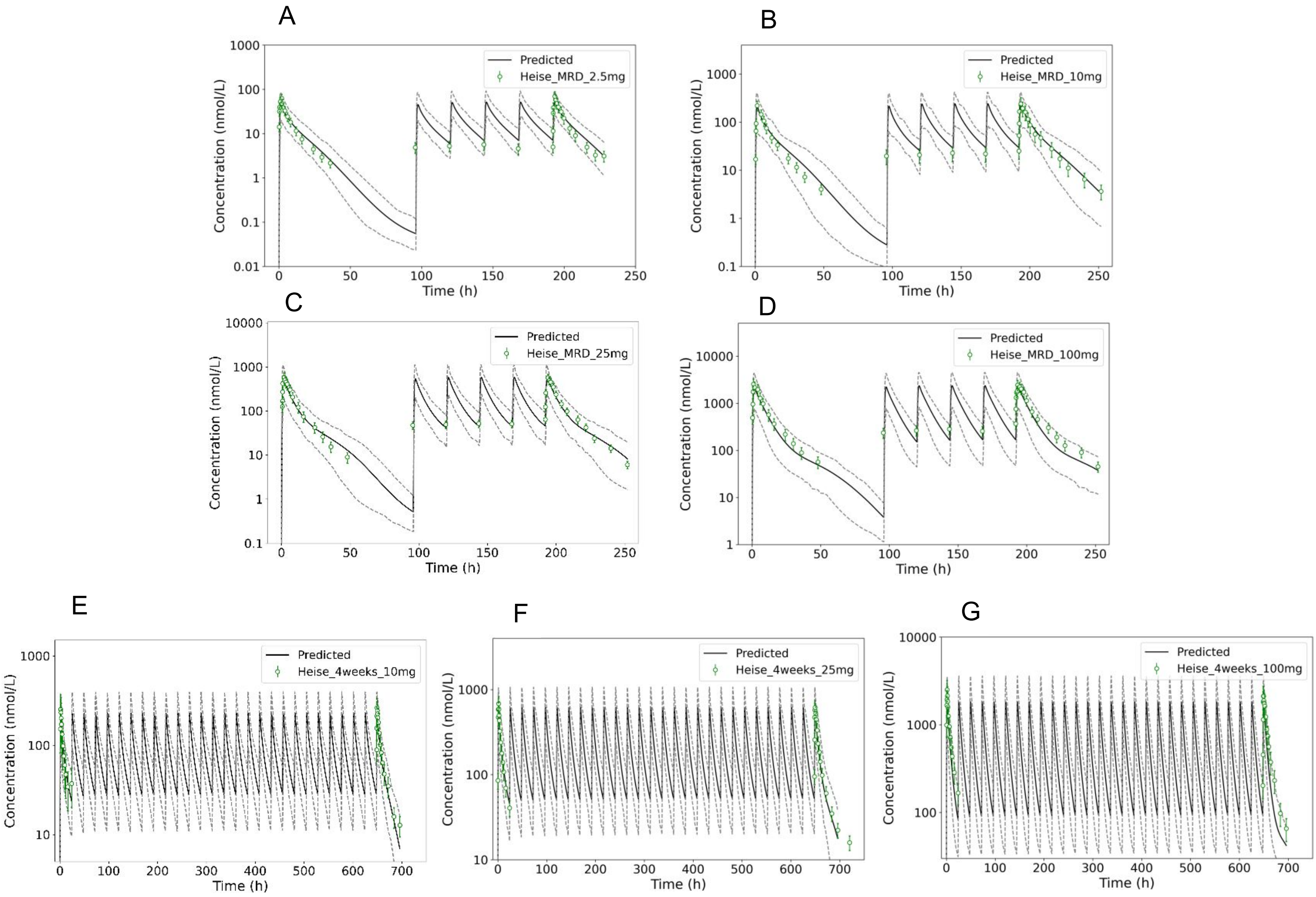

Figure S4 — PBPK-predicted plasma concentration–time profiles of EMPA under multiple-dose regimens, compared with observed clinical data from Heise et al. (2013). Panels A–D show the multiple rising dose (MRD) study with 2.5 mg, 10 mg, 25 mg, and 100 mg once daily (OD) for 8 days. Panels E–G show the 4-week study in patients with type 2 diabetes mellitus (T2DM) receiving 10 mg, 25 mg, and 100 mg OD. Solid black lines indicate predicted median concentrations; dashed lines represent 5th and 95th percentiles. Green symbols with error bars represent observed mean concentrations ± SD.

Table S6 — Comparison of pharmacokinetic parameters from individual and population simulations of children and adolescents (10–18 years) with observed adult data from the Seman et al., 2013 study and laffel study with a 10 mg dose.

**Individual simulations**							
**Healthy**				**Obese**			
Age	AUC_{inf} (nmol*h/L)	Cmax (nmol/L)	Tmax (h)	Age	AUC_{inf} (nmol*h/L)	Cmax (nmol/L)	Tmax (h)
10	3326.41	599.65	1.45	10	3255.44	458.05	1.5
11	3035.92	538.35	1.5	11	2952.8	402.94	1.5
12	2773.45	477.52	1.5	12	2687.42	350.59	1.5
13	2530.74	418.68	1.45	13	2453.92	303.31	1.5
14	2310.07	364.68	1.45	14	2250.84	262.54	1.5
15	2135.13	324.65	1.45	15	2090.47	233.34	1.5
16	2039.78	297.98	1.45	16	2008.03	214.44	1.5
17	1970.11	280.4	1.45	17	1945.58	202.46	1.5
18	1919.79	268.62	1.45	18	1899.82	195.62	1.5
**Population simulations**							
10-12	3112.24	546.21	1.44	10-12	2986.12	409.66	1.56
13-14	2424.12	374.47	1.58	13-14	2553.6	296.58	1.62
15-18	2014.28	274.43	1.54	15-18	1965.01	210.58	1.55
**Observed data**							
Obese children (Laffel et al., 2018)				14.5	1450	211	1.25
Adult (Seman et al., 2013)	1730	226	1.5	Adult (Seman et al., 2013)	1608.17	196.24	1.30

Table S7 — Comparison of pharmacokinetic parameters from population simulations of grouped children and adolescents (using PK-Sim) with observed data from single-dose clinical studies in adults (Seman et al., 2013).

	Dose	Age	Parameter	Obs	5%	95%	Pred	Pred/Obs
healthy	10mg	10-12yo	AUC(0-inf) (nmol·h/L)	1730 (377)	1992.96	4678.19	3112.24	0.56
			Cmax (nmol/L)	226 (46.0)	216.45	942.12	546.21	0.41
			Tmax (h)	1.5 (1.0–2.0)	0.8	2.26	1.44	1.04
		13-14yo	AUC(0-inf) (nmol·h/L)	1730 (377)	1413.71	3796.12	2424.12	0.71
			Cmax (nmol/L)	226 (46.0)	140.75	613.2	374.47	0.60
			Tmax (h)	1.5 (1.0–2.0)	1	2.5	1.58	0.95
		15-18yo	AUC(0-inf) (nmol·h/L)	1730 (377)	1129.59	3159.48	2014.28	0.86
			Cmax (nmol/L)	226 (46.0)	108.72	459.99	274.43	0.82
			Tmax (h)	1.5 (1.0–2.0)	1	2.25	1.54	0.97
obese	10mg	10-12yo	AUC(0-inf) (nmol·h/L)	3830 (825)	1872.61	4887.62	2986.12	1.28
			Cmax (nmol/L)	505 (130)	190.47	671.26	409.66	1.23
			Tmax (h)	2.1 (1.0–3.0)	1	2.5	1.56	1.35
		13-14yo	AUC(0-inf) (nmol·h/L)	3830 (825)	1467.77	4543.81	2553.6	1.50
			Cmax (nmol/L)	505 (130)	95.99	479.66	296.58	1.70
			Tmax (h)	2.1 (1.0–3.0)	1.05	2.5	1.62	1.30
		15-18yo	AUC(0-inf) (nmol·h/L)	3830 (825)	1059.7	2919.47	1965.01	1.95
			Cmax (nmol/L)	505 (130)	55.51	365.49	210.58	2.40
			Tmax (h)	2.1 (1.0–3.0)	0.95	2.5	1.55	1.35

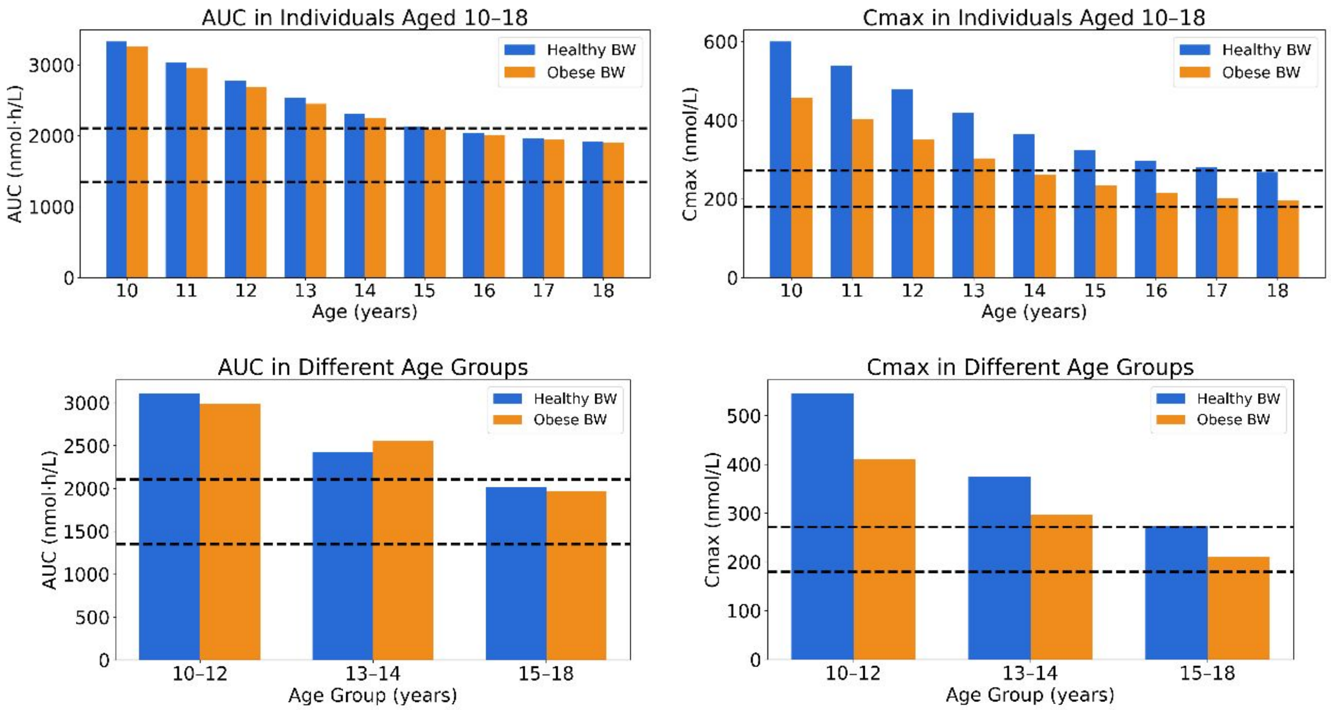

Figure S5 — Bar charts of EMPA AUC and Cmax, comparing individuals with healthy body weight and obesity in the 10-to-18-year age range. The top row of charts (AUC in 10-18-year-olds, Cmax in 10-18-year-olds) shows the individual values for each year of age, from 10 to 18. The bottom row of charts (AUC by age groups, Cmax by age groups) clusters this data into three age ranges: 10–12 years, 13–14 years, and 15–18 years. Dashed horizontal lines indicate observed reference values in adults (FDA, 2014).

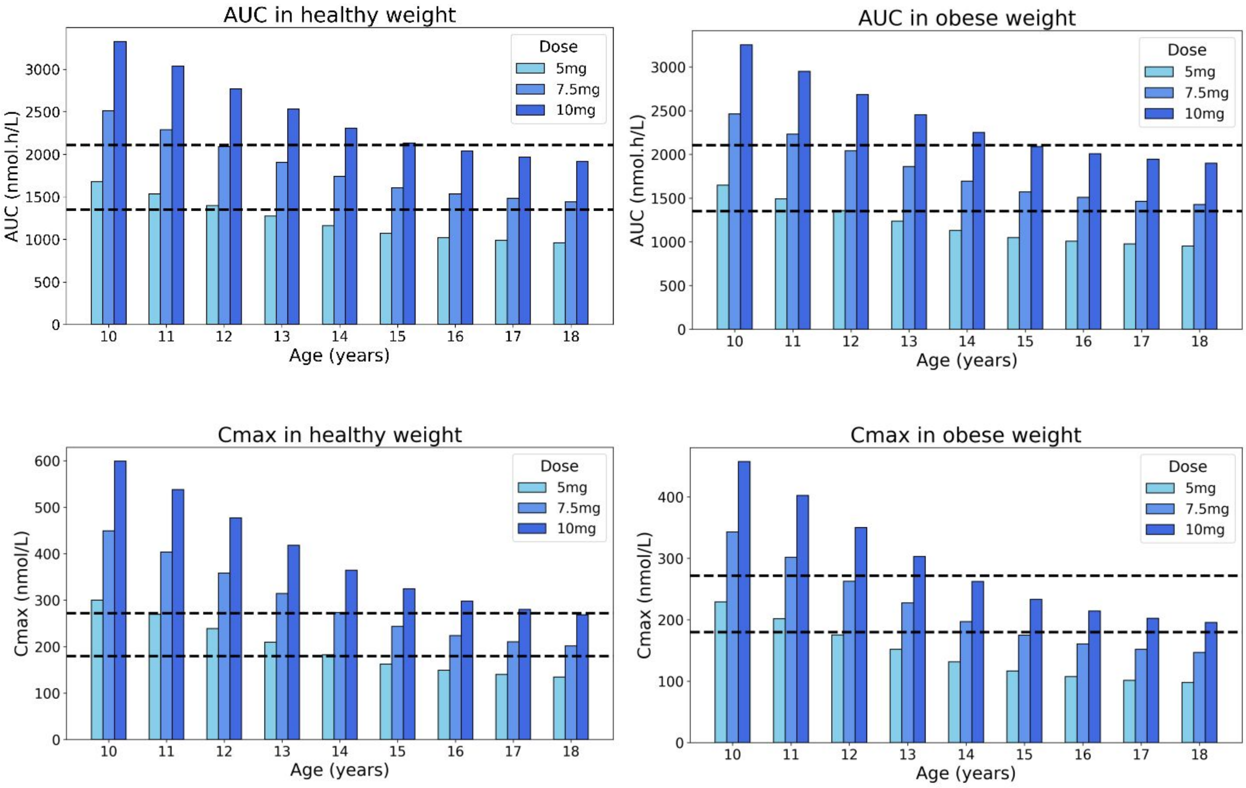

Figure S6 — AUC and Cmax across different ages (10-18 years) and dosages (5 mg, 7.5 mg, 10 mg), stratified by healthy and obese weight. The upper panels show AUC, while the lower panels present Cmax. Horizontal dashed lines indicate reference values in adults.

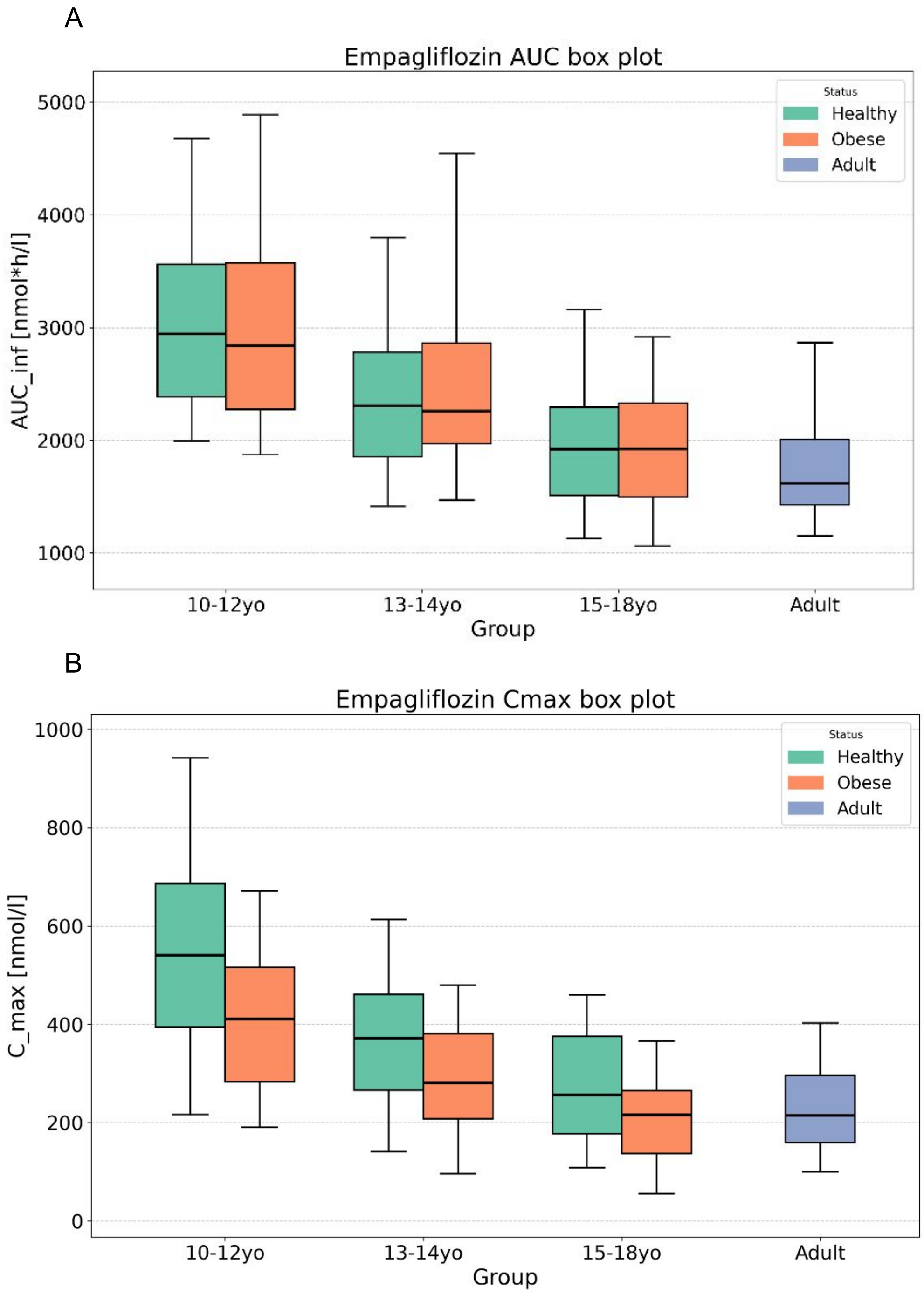

Figure S7 — Box plots of Empagliflozin AUC_inf and Cmax by pediatric age groups (10-12, 13-14, 15-18 years) and adults, stratified by health status (Healthy, Obese, Adult). (A) AUC; (B) Cmax.
